# Workload in the Surgical Center: perceptions, activities and time spent by nurses*

**DOI:** 10.1590/1518-8345.7549.4512

**Published:** 2025-03-31

**Authors:** Denilse Damasceno Trevilato, Fabiana Zerbieri Martins, João Lucas Campos de Oliveira, Rita Catalina Aquino Caregnato, Tarcísio Abreu Saurin, Ana Maria Müller de Magalhães

**Affiliations:** 1 Universidade Federal do Rio Grande do Sul, Porto Alegre, RS, Brazil.; 2 Hospital Moinhos de Vento, Porto Alegre, RS, Brazil.; 3 Hospital de Clínicas de Porto Alegre, Porto Alegre, RS, Brazil.; 4 Universidade Federal de Ciências da Saúde de Porto Alegre, Porto Alegre, RS, Brazil.

**Keywords:** Nurse’s Role, Workload, Perioperative Nursing, Intraoperative Period, Professional Competence, Surgicenters

## Abstract

to evaluate the workload perceived by operating room nurses and the time spent on the activities performed and to understand the activities that influence their perception.

sequential explanatory mixed methods study. The quantitative stage involved a survey of 34 nurses from five hospitals and measuring the time spent on each activity in the hospitals with the highest and lowest perceived workload, during 129 hours of observation. Semi-structured interviews were then conducted with 12 nurses. The quantitative data was analyzed descriptively and inferentially, and the qualitative data was submitted to content analysis and then integrated by connection.

there was a predominance of females (88.23%) and a mean age of 39±8.18 years among the nurses, with a moderate (r=0.502) and significant (p=0.002) correlation between age and perceived workload. Managerial activities spent more time and were identified as the most influential in the perception of the workload associated with the mental demand dimension.

the activities that have the greatest impact on the perception of workload include managing the sector and people. Direct patient care was identified as providing job fulfillment and purpose.

## Highlights

**(1)** Moderate and significant correlation between perception of workload and age. **(2)** The highest percentage of time measured was spent on managerial activities. **(3)** Mental demand scored highest in the perception of workload. **(4)** Direct patient care is a significant source of job fulfillment. **(5)** Nurses express a loss in the essence of care to the detriment of other activities.

## Introduction

The Surgical Center (SC) is a highly complex area in which the flow of information, materials, and people requires a significant coordination effort. In this scenario, the nurse’s role requires team leadership and communication skills, as well as carrying out various activities, such as managing people, materials, and the surgery schedule (surgical map), as well as direct patient care ^(1)^.

Nurses’ activities in the SC are mainly related to the organization of the sector and the surgical schedule, to allocate the scheduled procedures according to the physical structure and qualification of the team to best meet the needs of each situation ^(2)^.

Nursing workload can be defined as the amount of time spent performing a task, taking into account: the degree of complexity of caring for different patients; the level of knowledge and skills required of nurses to meet the different needs of patients; direct patient care; as well as the physical, mental and emotional effort of the professional to carry out nursing activities ^(3)^. Studies to determine workload by measuring the time spent on nursing activities have been carried out mainly in inpatient units ^(4 - 6)^ and Intensive Care Units (ICUs) ^(7)^.

However, workload also has a subjective dimension related to the way each nurse experiences their activities, and can be a predictor of the quality of patient care ^(8)^. One of the tools that can be used to identify perceived workload is the National Aeronautics and Space Administration - Task Load Index (NASA-TLX) scale, initially used to collect subjective information on the workload of pilots and air traffic controllers ^(9)^.

In health services, NASA-TLX has been used to assess the workload perceived by nurses in neonatal ^(8 , 10)^, adult ^(11 - 13)^, pediatric ^(8)^, pediatric inpatient ^(14)^, and emergency ^(15 - 16)^ units. In the SC, two studies were found: one that compared the implications of a new surgical instrumentation table design ^(17)^, and another ^(18)^ that evaluated the repercussions of the noise level in the operating room. As limitations, these studies did not consider all the activities carried out by the nursing team and did not provide a systemic assessment of the workload in the SC supported by multiple sources of evidence, both qualitative and quantitative, as in this proposed mixed-method study.

These considerations and the specificity of work in the SC have demonstrated the need to make sensitive efforts to capture the workload perceived by nurses in this sector and the activities they carry out. Given this, the objectives of this study were: to evaluate the workload perceived by operating room nurses and the time spent on the activities performed and to understand the activities that influence their perception.

## Method

### Study type

This is a three-stage sequential explanatory mixed-methods study, with greater weight given to the quantitative approach (QUAN➝ qual) ^(19)^. The designs of each stage were as follows: 1) survey; 2) observational study; and 3) exploratory study. In the first two quantitative stages (QUAN), the points to be explored in the third (qual) were identified. The mixed method was chosen due to the possibility of integrating qualitative data with quantitative data, to complement and enrich the understanding of the phenomenon under study, in the quest to clarify the activities that influence the perception of workload by nurses in the SC.

The Strengthening the Reporting Observational Studies in Epidemiology (STROBE) ^(20)^, Consolidated Criteria for Reporting Qualitative Research (COREQ) ^(21)^, and Mixed Methods Appraisal Tool (MMAT) ^(22)^ guides were used to conduct and report this research, seeking transparency and reproducibility in the investigation.

### Study site

It was carried out in the SC of five large public and private hospitals in Porto Alegre, Rio Grande do Sul, Brazil.

### Study length

Between March and September 2023, sequentially, according to the planned stages.

### Population

Nurses working in the SC of the five institutions during the collection period, totaling 60 nurses.

### Selection criteria

Inclusion criteria: nursing assistants working in SCs with eight or more operating theaters and with a minimum of six months working in the SC.

Exclusion criteria: nurse managers or nurses on leave for any reason during data collection.

### Definition of participants

In the first stage (survey), a non-probabilistic convenience sample was used, consisting of 34 (56.67%) completed questionnaires. For the second observational stage, the hospitals with the highest (Hospital B) and lowest (Hospital D) scores for perceived workload by the nurses who responded to the survey were selected. Fifteen six-hour work shifts were observed in each of the two hospitals, with one nurse/observer/shift, totaling 129h22min, with a total of 30 shifts and 15 nurses observed. In the third, exploratory stage, 12 nurses who worked in the two hospitals selected for the observational stage were interviewed, according to interest and availability, with the sample delimitation being obtained by data saturation ^(23)^.

### Study variables

The variables were: perceived workload score using the NASA-TLX scale, activities carried out by nurses, time spent on these activities, sociodemographic variables, and the work environment.

### Instruments used to collect information

In the first stage, an online form was used via the Research Electronic Data Capture (REDCap^®^) platform ^(24)^ containing: a Free and Informed Consent Term (FICT); extraction of sociodemographic and work environment data (age, gender, work shift, monthly workload, number of operating rooms, number of nurses on shift); and the NASA-TLX scale ^(9)^, an instrument translated and validated into Portuguese ^(25)^.

In the NASA-TLX scale, the six dimensions (Mental Demand, Physical Demand, Temporal Demand, Performance, Effort, and Frustration Level), paired together, resulted in 15 combinations (for example: Mental Demand X Physical Demand), with the one that contributed most to the work being chosen in each pair. The six dimensions were then scored separately on a continuous scale, with the score ranging from one to 100, representing the level of influence on the workload ^(9 , 22)^.

The perceived workload was calculated individually using the NASA-TLX scale, following four steps described by Terra ^(26)^. Firstly, we counted how many times each item was considered the most influential among the six dimensions (for example, in how many of the 15 comparisons effort was preponderant). Then, to calculate the weight of each dimension, the number of times the item was chosen was divided by 15, which is the maximum number of pairings. In the second step, the dimension score was obtained by multiplying the dimension weight by the level of influence, which ranges from one (little influence) to 100 (a lot of influence), except for the performance dimension, whose value was inverted, since the greater the influence, the better. In the third step, the values were rescaled so that the scores for each dimension were parameterized from zero to 10, dividing the value obtained for workload by the maximum possible value (5/15=0.33), and this result by 10. Finally, the overall workload index for each individual was obtained from the sum of the six dimensions, also rescaled from zero to 10. Currently, there is no solid evidence on the reference values and classifications of the scores, so it is considered that the higher the score, the greater the perception of workload.

The software TrackingTime® ^(27)^ was used to measure activity time, with an initial list of 23 activities carried out by SC nurses in Brazil, identified in a scoping review ^(28)^. During the observation, ten activities were added, and after regrouping according to the frequency and similarity of the work processes, a result of 26 observed items was obtained. During data collection, the stopwatch was activated at the start of the activity and deactivated at the end, pausing and restarting at each interruption.

The questions addressed in the third stage interviews were conducted to explore the results of the first and second stages using a structured script, with questions related to the activities that most impact the perception of workload, the activities recognized as important but which they are unable to carry out during their work shift, as well as the activities that bring the most job fulfillment.

### Data collection

The quantitative stages took place between March and August 2023 and were conducted through an invitation sent via e-mail containing the link to the form and instructions on how to fill in the instrument. After analyzing the results of this stage, the two SCs for the observational stage were defined. The collection team for this stage was made up of the study’s main researcher, a scientific initiation scholarship holder, and two volunteer nurses from the research group. A tutorial was prepared on the use of the TrackingTime® ^(27)^ application, which had been previously tested by the researchers for the observation, but without a pilot test with the nurses in the real practice scenario. At the start of their shift, the nurses were invited to be observed while carrying out their activities, throughout the six-hour period that comprised their shift at the SC.

The third stage took place between August and September 2023 and was conducted by the main researcher. Participants were given the letter “P” followed by a numeral. The interviews were recorded, transcribed, and returned by email for validation by the respondents, with a deadline of one week. Of the participants, one person returned the transcript fully validated, another requested adjustments, and the others did not respond within the established timeframe. This process of returning the transcripts allowed the research team to reflect on the importance of involving the respondents in validating the information, even if not all of them returned it, highlighting it as a positive point in the search for rigor and transparency in the research.

### Data processing and analysis

The quantitative data was organized in a Google^®^ spreadsheet ^(29)^ and imported into the Statistical Package for the Social Sciences (SPSS) software ^(30)^, version 25.0, and analyzed descriptively and analytically. Perceived workload was measured using the NASA-TLX scale and was calculated individually according to the four steps described in a previous study ^(26)^, with the final score being rescaled to a scale of one to 10.

The Shapiro-Wilk (S-W) test was used to assess the normal distribution of the numerical data. Student’s *t*-test, ANOVA, and Pearson’s bivariate correlation were used to relate the sociodemographic variables to the NASA-TLX scale score, adopting a significance level of 5% (p≤0.05).

The frequency and average time spent on the activities were classified according to the three main areas of activity for nurses: management, care, or teaching/research, as listed in the scoping review ^(28)^. The time used for breaks/personal care was also calculated.

The qualitative data was submitted to Bardin’s Content Analysis ^(31)^, respecting the stages of pre-analysis, exploration of the material, treatment of the results, and interpretation. NVivo® ^(31)^ software, version 14.23.2, was used as an auxiliary tool in the process of organizing and systematizing the data. In total, 9h23min of recordings were obtained, resulting in 39 pages of transcript with around 20,300 words. To reach a consensus during the coding and categorization stages of the results, meetings were held between the researchers, ensuring consistency in the interpretation of the data.

Initially, the interview transcripts were imported into NVivo® ^(32)^ software, which allows quick and precise navigation through the corpus, as well as the possibility of making notes directly on the text. This process made it easier to break down the recording units, group them, and regroup them. In the categorization stage, relationships were established between the units of meaning identified in the coding, bringing together the elements that could be combined and/or classified, which gave rise to the categories. From these categories, new understandings of the phenomenon under study emerged, enriching the analysis.

The data was integrated by connecting the three stages, presenting meta-conferences based on the analysis of the integrated results, as well as a joint display. The joint display consists of clearly visually representing the quantitative and qualitative aspects of a mixed methods study. The iterative process of developing these representations can improve understanding of the integrative analysis by including diagrams based on the study’s findings ^(33 - 34)^.

### Ethical aspects

The study was approved by the Research Ethics Committee (REC), Certificate of Submission for Ethical Appraisal (CAAE): 62257722.8.0000.5347.

## Results

Among the 34 nurses who returned the survey, it was found that four (12%) belonged to each of Hospitals A, C, and E, 10 (29%) to Hospital B, and 12 (35%) to Hospital D. The sample was predominantly female (n=30; 88.23%); with a mean age of 39±8.18 years; 12 (35.29%) worked the afternoon shift; and 24 (70.58%) worked 180 hours a month.

The number of operating rooms in the SCs varied between 12 and 18, with an average of 15±2.83 rooms. There were, on average, three ±1.31 nurses on each shift in the SCs of each hospital, and a median of 24 (12;36) nursing technicians under their supervision. The number of nurses per shift varied between one and five, and the number of technicians varied between five and 60.

The mean overall index of the NASA-TLX scale ranged from 6.05±1.00 to 7.34±1.00, with no significant difference between the hospitals (Table 1).

**Table 1 -:** Scores for the dimensions and overall index of the NASA-TLX scale, as perceived by Surgical Center nurses (n = 34). Porto Alegre, RS, Brazil, 2023

**Hospital**	MD*	**PD** ^†^	**TD** ^‡^	**PE** ^§^	**EF** ^||^	**FR** ^¶^	**Global index** ^‡‡^
	m** (sd) ^††^	**m** ^**^ **(sd)** ^††^	m** (sd) ^††^	m** (sd) ^††^	**m** ^**^ **(sd)** ^††^	m** (sd) ^††^	**m** ^**^ **(sd)** ^††^
A	6.00 (2.40)	0.81 (1.62)	3.00 (2.54)	0.91 (0.48)	4.61 (2.02)	7.99 (2.17)	7.00 (1.22)
B	5.40 (2.22)	0.55 (1.17)	3.96 (2.3)	2.14 (1.85)	5.70 (3.48)	6.69 (3.02)	7.34 (1.00)
C	4.10 (2.64)	2.30 (3.83)	4.92 (2.42)	1.32 (1.01)	5.71 (3.75)	4.60 (3.72)	6.90 (1.32)
D	5.41 (2.03)	1.39 (1.30)	4.77 (2.86)	1.15 (1.27)	4.68 (2.70)	3.10 (2.95)	6.15 (1.08)
E	5.49 (2.55)	1.05 (0.71)	2.86 (1.63)	1.48 (1.33)	6.64 (2.40)	2.70 (4.88)	6.05 (1.00)
Total	5.33 (2.17)	1.14 (1.69)	4.12 (2.47)	1.47 (1.40)	5.32 (2.89)	4.86 (3.62)	6.68 (1.16)

*MD = Mental Demand; ^†^PD = Physical Demand; ^‡^TD = Temporal Demand; ^§^PE = Performance; ^||^EF = Effort; ^¶^FR = Frustration Level; **m = Mean; ^††^SD = Standard Deviation; ^‡‡^ANOVA (p=0.111)

**Figure 1 -:**
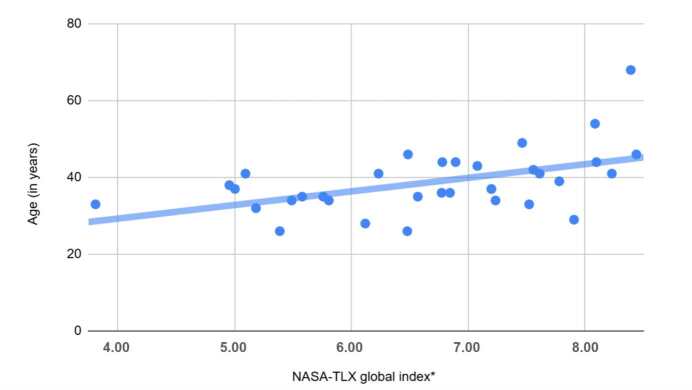
Global index of the NASA-TLX* scale according to age (n = 34). Porto Alegre, RS, Brazil, 2023

There was no significant correlation between the following variables and the overall index of the NASA-TLX scale: sex (p=0.789), work shift (p=0.057), monthly workload (p=0.095), number of nurses on shift (p=0.318), number of rooms (p=0.080), number of operating rooms under their responsibility (p=0.564), and the number of nursing technicians under their direct supervision (0.245). Age showed a moderate (r=0.502) and significant (p=0.002) correlation with the overall index of the NASA-TLX scale (Figure 1).

*NASA-TLX = National Aeronautics and Space Administration - Task Load Index

Measuring the activities carried out in the two selected hospitals resulted in a total of 129h22min of observation, 60h16min in Hospital B, and 69h6min in Hospital D. There was a predominance of time dedicated to management in Hospital D (p<0.001) and assistance in Hospital B (p=0.004), with a significant difference between them (Table 2).

**Table 2 -:** Distribution of time according to the activities carried out by Surgical Center nurses (n = 15). Porto Alegre, RS, Brazil, 2023

**Activity**	T* HB ^†^ **h** ^§^ **/m** ^||^ **(%)**	T* HD ^‡^ **h** ^§^ **/m** ^||^ **(%)**	T* total h ^§^ **/m** ^||^ **(%)**	**p-value** ^¶^
**Management**	25:49 (42.84)	44:45 (64.76)	70:34 (54.55)	**<0.001**
A1. Planning the roster for the other day/shift	05:42 (9.46)	06:05 (8.80)	11:47 (9.11)	
A2. Resolving administrative issues	02:56 (4.87)	07:52 (11.38)	10:48 (8.35)	
A3. Team management (sizing, time off, vacations)	00:53 (1.47)	07:16 (10.52)	08:09 (6.30)	
A4. Managing the roster (slots, urgency, redeployment)	02:10 (3.60)	05:28 (7.91)	07:38 (5.90)	
A5. Shift handover	05:18 (8.79)	02:14 (3.23)	07:32 (5.82)	
A6. Organizing the SC**/supervising the rooms	01:33 (2.57)	05:39 (8.18)	07:12 (5.57)	
A7. Supervising the nursing team	00:31 (0.86)	02:44 (3.96)	03:15 (2.51)	
A8. Instruments (lack or failure)	02:06 (3.84)	00:47 (1.13)	02:53 (2.23)	
A9. Assisting with room cleaning/assembly	00:05 (0.14)	02:36 (3.76)	02:41 (2.07)	
A10. Equipment (faulty or missing)	01:31 (2.52)	00:47 (1.13)	02:18 (1.78)	
A11. Map/round	01:23 (2.30)	00:00 (0.00)	01:23 (1.07)	
A12. Input management	00:09 (0.25)	00:47 (1.13)	00:56 (0.72)	
A13. Computer Troubleshooting	00:00 (0.00)	00:21 (0.51)	00:21 (0.27)	
A.14. Anesthetist warning	00:09 (0.25)	00:08 (0.19)	00:17 (0.22)	
A15. Recording occurrences and incidents	00:06 (0.17)	00:06 (0.14)	00:12 (0.15)	
A16. Updating the other day’s roster for the Material and Sterilization Center	00:08 (0.22)	00:00 (0.00)	00:08 (0.10)	
A17. Transplant notice	00:07 (0.19)	00:00 (0.00)	00:07 (0.09)	
**Assistance**	30:32 (50.66)	17:17 (25:01)	47:49 (36.96)	**0.004**
A18. Patient assessment and documentation of care provided (Nursing process)	17:33 (29.12)	04:01 (5.81)	21:34 (16.67)	
A19. Monitoring induction of anesthesia and assistance with difficult intubation	06:09 (10.2)	02:32 (3.67)	08:41 (6.71)	
A20. Direct patient care in the operating room	02:19 (3.84)	04:23 (6.34)	06:42 (5.18)	
A21. Surgical positioning	03:20 (5.53)	02:33 (3.69)	05:53 (4.55)	
A22. Nursing procedures (puncture, probing)	01:09 (1.91)	03:18 (4.78)	04:27 (3.44)	
A23. Transfer of care (Intensive Care Center/Unit)	01:04 (1.77)	01:55 (2.77)	02:59 (2.31)	
A24. Care for intercurrences	00:00 (0.00)	00:30 (0.72)	00:30 (0.39)	
**Teaching/research**	00:04 (0.11)	00:47 (1.13)	00:51 (0.66)	1.00
A25. Team training	00:04 (0.11)	00:47 (1.13)	00:51 (0.66)	
**Personal care**	03:51 (6.39)	06:17 (9.09)	10:08 (7.83)	0.024
A26. Personal break	03:51 (6.39)	06:17 (9.09)	10:08 (7.83)	

*T = Time in hours and minutes; ^†^HB = Hospital B; ^‡^HD = Hospital D; ^§^h = Hours; ^||^min = minutes; ^¶^Mann-Whitney U test; **SC = Surgical Center

The five most prevalent activities in the hospitals observed, which accounted for 47.14% of the time measured, were: patient assessment and documentation of the care provided (nursing process), planning the schedule for the next day/shift, resolving administrative issues, monitoring anesthetic induction and team management. The 10 least frequent activities accounted for 5.45% of the total time measured: equipment (failure or lack of equipment), map/round beating, managing supplies, resolving computer problems, warning the anesthesiologist, recording occurrences and incidents, updating the next day’s schedule for the materials and sterilization center, warning/notifying the transplant, attending to intercurrences and training the team. The time used for personal breaks corresponded to 7.83% of the nurses’ working days.

**Figure 2 -:**
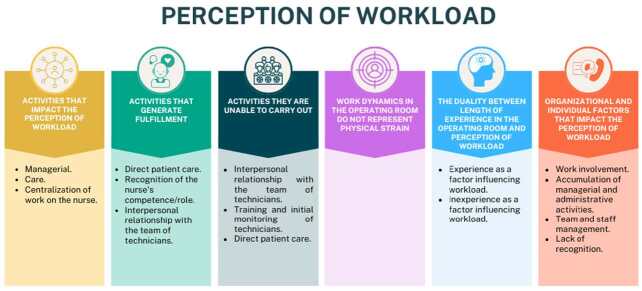
Factors influencing the perception of workload by Surgical Center nurses (n = 12). Porto Alegre, RS, Brazil, 2023

From the analysis of the interviews concerning the activities that most influenced the perception of workload, six categories emerged, represented by different colors, and 15 subcategories, as shown in Figure 2. The categories described below are illustrated with excerpts from the interviews.

### Activities that impact the perception of workload

Nurses associate managerial activities with an increased perception of workload, especially concerning managing the surgical schedule, because *[...] even to set up an operating room, it’s not just putting the room there, I have to think about the team that’s going to be there, who’s going to attend, if there’s going to be equipment, if there’s going to be material, and all of this requires a lot of planning [...] (P2)* and nursing professionals, which involves the reassignment of workers and the organization of the sector to meet the SC’s demand., *[...] organizing the daily roster, not of surgeries, but of people, [...] I have to remember that someone is on sick leave, someone isn’t coming. Apart from looking at a schedule, I have to look at several schedules to produce one [...] (P10)*.

Mental overload is impacted by conflict management, *I manage situations with all these teams. The surgical team, the anesthesia [team], my nursing team, and with patient care [...] (P2)*, can lead to frustration and strained relationships between teams, *[...] of us feeling unappreciated, belittled, demeaned, various negative things to the detriment of a conflict that you can’t manage (P3)*.

The inability to be present at the beginning of all surgical procedures and unsuccessful complications were identified as care activities that influenced the perception of workload, *[...] maybe it’s because I’ve had this whole nursing process, where we really have to do all this systematization, we have to assist in the room [...] positioning, helping with induction, in short, organizing surgical equipment, surgical materials, [...] (P2), [...] the most frustrating thing is that you have four rooms and, when you see [...] they’ve already gone in and you haven’t managed to keep up with the start (P5), and [...] when we can’t reverse a cardiac arrest, for example, when you attend to a baby and [...] you don’t succeed (P2).*

The centralization of information and attributions in the figure of the nurse was highlighted by the participants, because *[...] it seems like it’s all our fault, because the anesthesia isn’t working, because the room has been blocked, because the material hasn’t come down from the CMS [Central Material and Sterilization], it’s all on the nurse’s back, and then, for the technician, it seems like the flows, nothing works, that we don’t know what’s going on (P5)*.

### Activities that generate fulfillment

Direct patient care is mentioned as the biggest fulfillment factor, *[...] when I take care of the patient directly in the room [...], look at them, hold their hand, position them after they’ve been anesthetized with the utmost care. (P3)*, and take part in a transplant, *[...] a surgery of this complexity and with such a huge impact on an entire family, which is to remove a piece of the father’s liver [and implant it] in the child. (P3)*.

Recognizing the competence and importance of the nurse’s presence in the operating room: *[...] I think it’s great when someone says: “I’m glad the nurse is in the room”. Because you feel that people are happy that you’re there, that if they need you, they know you’ll be able to help them (P3)*, and the interpersonal relationship with the team of nursing technicians was pointed out as activities that generate fulfillment, through *[...] a return on what they’ve asked me for. Even if it’s time off. Sometimes I’ve managed to give that technician feedback in good time, but I don’t give a positive response to the time off, sometimes even a negative one (P4)*.

### Activities they can’t do

While the interpersonal relationship with the team brings fulfillment, it is also recognized as an activity with insufficient time to devote to it: *[...] giving more attention to the personal side of your nursing team [...] is something I miss. (P1) Being able to talk more with the nursing technicians about trivial things in life. Getting to know their history better (P3).*

Training and initial support for the nursing technician are compromised by the demand for work, *[...] training, mentoring a new employee, who is very flawed, we leave him out in the cold, and he picks it up on his own [...] (P9)*, can be detrimental to the employee’s development and assessment throughout the year. The need to improve teamwork, coupled with caring for others, is identified vehemently at times *[...] not seeing people as people for lack of time, [...] this is not a priority because we have other demands (P12)*, This is an important factor in establishing trusting and quality bonds.

Due to the dynamics of the SC, nurses are often unable to get involved in direct care at the start of the procedure, *[...] being there when the patient was being intubated, positioned, so when you enter the room and the surgery has already begun (P5)*, which could be a factor of frustration, since this was also pointed out as one of the activities that generates the most fulfillment.

### Work dynamics in the SC do not represent physical strain

Nurses do not recognize physical demands as influencing their perception of their workload, and it is more frequent in large and complex surgeries, such as transplants, *[...] I don’t feel physically tired, except when I have to attend to a transplant and I stay inside the room exclusively. In that specific situation, I leave physically tired, but feeling so fulfilled [...] (P3)*.

The physical strain is pointed out in the condition of taking on several operating theaters, when there is a reduction in staff, as expressed by *[...] there are fewer nurses, the [...] physical demand is much greater too (P4)*. Another participant said that, as much as the SC represents *a very large area here, [...] you don’t have to go to other places, [because it has] a delimited physical area [...] Nursing procedures [...] are not tiring [activities] (P6)*.

### The duality between length of experience in SC and perception of workload

Experience can contribute to a greater perception of the workload, as they have a better view of the needs of the sector and the institution, and greater involvement in processes and committees, due to their qualifications and recognition by the team as a reference in resolving certain situations, as expressed in the following excerpts: *[...] the people who have been there the longest end up being a reference, I don’t mean specialties, but for some functions (P1) [...] the more experience we have, the more responsibilities we have, [...] I realize that not only age but length of service, and contribution within the sector (P5)*.

On the other hand, some more experienced professionals reported a lower perception of the workload, because *over the years, you learn [...] not to let this influence your work so much (P1)*, for having already reached a *level of relationship with the surgical team, I don’t mean intimacy, but trust, the credibility of working with them (P3)*.

Inexperience was pointed out as a factor that could contribute to a lower perception of the workload by the physical disposition *because they have energy and drive (P11)*, and *a high capacity to adapt to situations (P12)*, or because they don’t know the routines, the problems *and don’t foresee avoidable occurrences, or as a factor that can increase this perception because they don’t know how to deal with the unexpected or [...] don’t have enough expertise to deal with complications (P2)*.

### Organizational and individual factors that impact workload perception

The level of personal involvement as well as the non-involvement of colleagues increases the feeling of work overload, *[...] because if you’re a person who knows how to take your place, who knows the difference you make, you’ll get involved in more work fronts (P3)*.

The accumulation of managerial and administrative activities leads to a detour of the nurse’s care role, as they take on demands that could be carried out by an administrative team: *[...] we’re losing what used to be the essence of care to do other things, so our load is increasing as a result. The flows, the processes that aren’t well aligned; they usually fall to the nurse. [...] the nurse doesn’t know how to say no either, and you’re not going to keep the patient waiting, you’re going to receive material when you’re not supposed to, you’re going to go to the pharmacy to get something [...], you’re going to look for an invoice from another surgery that nobody has seen [...] (P5)*.

The management of the team and the adequacy of the staff were mentioned as a factor contributing to an increase in the perception of workload in one of the hospitals, according to the report: *[...] he lack of staff certificates, the sizing, the demand for nurses, the emergency room, having more complex surgeries, and us not having an increase in the staff (P7)*. On the other hand, at the other hospital, it was reported that the completeness of the nursing staff contributed to a lower perception of the workload *because [...] staff sizing has improved a lot (P8) [...] because, as much as there are a lot of things to do, today we have more nurses. When I was on my own, I thought my workload was much greater because I had to do vacations, the monthly roster, I had to do everything [...] and today I’m responsible for a roster, I still can’t get used to it (P10)*.

The lack of recognition expressed by nurses when they said that becoming just another number can contribute to making their efforts invisible because *if you become just another common denominator, and sometimes because of the high demand, you sometimes can’t be so apparent that you get recognition. So this lack of recognition sometimes generates frustration (P12)*.

**Figure 3 -:**
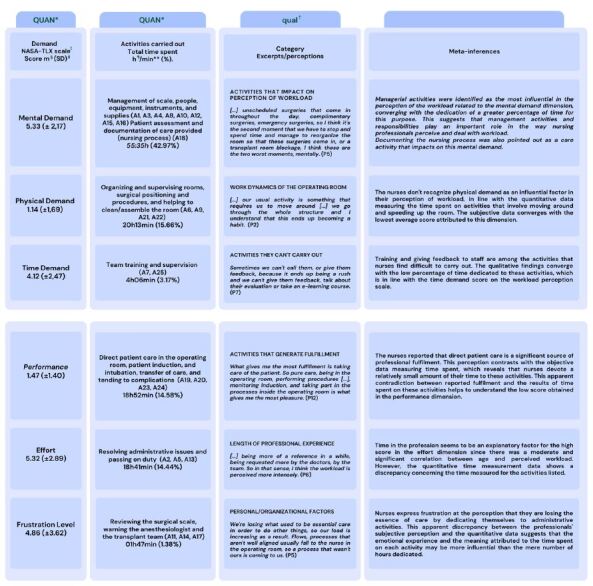
Integration of data from the global index of the NASA-TLX scale, time spent performing activities, and the perception of workload by Surgical Center nurses. Porto Alegre, RS, Brazil, 2023

The joint display and the integration of the data were presented in a joint display following the connection proposal, where the qualitative findings seek to explain and complement the quantitative data (Figure 3).

*QUAN = Quantitative; ^†^qual = Qualitative; ^‡^NASA-TLX = National Aeronautics and Space Administration - Task Load Index; ^§^m = Mean; ^||^SD = Standard Deviation; ^¶^h = Hours; **min = Minutes

## Discussion

Mental demand was the dimension that obtained the highest score on the NASA-TLX scale, reflecting the extent to which nurses perceived this dimension as the most challenging. The reasons why the nurses perceived the influence of this dimension on their workload became clearer with the data from the interviews, which reported that among the most influential activities were: planning staff schedules and the flow of the surgical schedule, managing the sector, and carrying out the patient assessment and documentation; these were also the activities that demanded most of their time. These findings converge with other studies, which have pointed out that the managerial role played by nurses in the SC focuses mainly on the organization of the sector and the dynamics of workflows ^(2 , 28 , 35)^.

In the SCs observed, unscheduled activities were mainly related to room relocations due to surgical delays and/or cancellations, relocation of staff to adapt rooms, and blocking for emergency care and transplants. The unpredictability and difficulty in maintaining planning, as well as the accumulation of multiple and concomitant activities centered on the professional nurse, can overload these workers ^(35 - 37)^. A study conducted in hospitalization units highlighted that unscheduled activities are a predictor of mental workload ^(38)^, which converged with the nurses’ perceptions, reflected in the SC’s dynamic characteristics.

The prevalence of time spent on patient assessment and documentation (16.67%) was lower than the result found in an American study, in which nurses spent 27% of their time on health records ^(39)^. The perception that this activity has an impact on mental demand converged with the results of studies in the Netherlands ^(40)^ and Italy ^(41)^ in which more than half of the nurses pointed to documentation as one of the causes of their perception of high workload, which can be associated with pressure and the substantial increase in time spent on this activity ^(38 - 39)^. To mitigate the impact on workload, it has been pointed out that the documentation process should be carried out in a more integrated way, with equipment close to the patients. This contributes to the recording taking place almost concurrently with the care ^(39)^, which can mitigate memory bias and, consequently, reduce the pressure on nurses to be assertive in their records.

The dimension with the lowest score on the NASA-TLX scale was physical demand, which indicates that the nurses did not recognize the influence of this dimension on the perception of workload, compared to the other dimensions, suggesting an ability to adapt to the physical demands of daily life in the SC. The low score on the NASA-TLX scale and the lower percentage (15.66%) of time spent on activities such as organizing the ward and surgical positioning suggested that the nurses felt adapted to the physical structure of the ward and the tasks that required physical effort. This finding contrasts with the results of a study that highlights the consequences of physical demand on nurses’ health since a previous study found that 77.7% of operating room nurses reported work-related musculoskeletal disorders ^(42)^. Furthermore, in a systematic review and meta-analysis, lumbar problems were the most common among perioperative nurses, with a prevalence of 62% in 19 studies ^(43)^.

When the time demands were analyzed in conjunction with the information from the interviews, it emerged that the nurses felt they needed to devote more time to supervising the team and establishing a bond with them, i.e. accompanying the new nursing technicians at the institution, reviewing and standardizing care activities in the operating room, as well as promoting training for the team. These findings reinforce the need to invest in continuing education, even in the face of the intense routine of the sector, as updates and training for the nursing team are considered essential for the quality of care provided ^(1)^.

The percentage of time spent (14.58%) on activities related to presence in the operating room, emotional support for the patient, and direct care, converged with the low score for the performance dimension on the NASA-TLX scale, demonstrating the nurses’ lesser involvement in these activities. The qualitative findings contributed to understanding these results when nurses expressed their desire to be closer to patient-centered care. Few studies have advanced in relation to the time dedicated to direct patient care activities in the operating room. A previous study carried out in a Brazilian setting, showed that nurses considered their presence in the operating room to be important, even in less complex procedures ^(44)^.

The participants reported supervising an average of five operating rooms for each nurse and considered this number to be adequate given the demands of their activities. It is known that adapting the nursing team to the workload is a challenge, and its importance is related to patient safety since an insufficient number of staff inevitably increases the workload and is a factor that can lead to the occurrence of adverse events and negatively influence the quality of care ^(45 - 46)^. Brazil’s Federal Nursing Council ^(47)^ recommends deducting the number of nursing hours required in the SC by the volume of procedures segregated into sizes, providing for one nurse for every three (elective) operating rooms, also considering the need for an exclusive nurse for emergency surgeries or according to their complexity. It is possible to infer that the low number of nurses (i.e. the professional with the highest level of training) working with patients is related to the low number of nurses working shifts. However, the findings of this study led to reflection on the adequacy of the workload, not just based on numbers, but related to the characteristics of the institution in terms of the specific procedures carried out (such as robotic surgery and transplants), as well as the training and experience of the professionals.

Among the personal factors, the positive correlation between age and perceived workload demonstrates the tendency for perceived workload to increase as age increases. Qualitative data contributed to clarifying these findings, where more experienced professionals were highlighted as a reference in the team, both in terms of knowledge and problem-solving expertise. In contrast to this, a systematic review and meta-analysis of results with the same instrument identified a greater perception of workload among inexperienced nurses, indicating that this may be related to a lack of knowledge of the nursing process and insecurity in resolving adverse situations ^(48)^.

Organizational factors can have an impact on the perception of workload, even though little time was spent on activities related to reviewing the surgical schedule for the following day, and communication with the anesthetic and transplant team, which were pointed out by the participants as activities that took nurses away from care. The identification and establishment of administrative tasks that can be delegated under the supervision of nurses need further study, including the use of computerized resources or artificial intelligence, as proposed by a German study protocol with the aim of testing and evaluating the acceptance of users (nursing staff, service staff, and patients) in the implementation of a robotic-digital system to relieve the overload of activities in the nursing service ^(49)^.

Nurses often associate managerial functions with an increase in perceived workload, especially when the focus is on managing surgical schedules rather than direct patient care. This view, however, is not just about mental overload, but seems to reveal the deeper aspects linked to nursing training and values, historically prioritizing care activities to the detriment of managerial, teaching, and research skills. The dichotomy between direct patient care and service management reflects a cultural construction that places greater value on care practices to the detriment of the leadership positions held by nurses. This condition is worrying, as it can lead to the development of nurses’ managerial competence, and service management can be seen as dispensable. This scenario could have serious consequences for the profession, considering that the WHO itself ^(50)^ has highlighted the positive impact of the leadership of nurse managers in services, but has also pointed out that this presence is still limited in number and hinders the implementation of health policies on a global scale.

A limitation of this study is the number of participating professionals and institutions, which may restrict the generalizability of the results. The specific characteristics of the participants and the variety of practices and approaches may not reflect the entire population in question. We, therefore, suggest expanding the diversity and representativeness of the sample in future studies to strengthen the robustness and applicability of the conclusions reached.

The results of this study provide new insights into how the activities performed and the time spent by nurses can have repercussions on the perception of workload. The analysis of time spent on different activities has the potential to contribute to the rationalization of time and resources, as well as the appropriate sizing of teams. An in-depth understanding can help develop strategies to optimize the distribution of tasks, improve operational efficiency, and promote a more balanced and satisfactory working environment for these professionals. The study can support discussions by professional associations and specialty societies about the duties of SC nurses and their greater involvement in direct patient care, which will lead to a review of team composition.

## Conclusion

When evaluating the perceived workload and the time spent on the activities carried out by SC nurses, the highest score was given to the mental demand dimension, with a predominance of activities related to managing the sector and people, as well as activities related to patient assessment and documentation. Although management tasks demand a preponderant portion of these professionals’ time, the direct connection with patient care during surgical procedures, even to a lesser extent, rewards them by providing job fulfillment and purpose in their work. This duality between efficient management and direct patient care highlights the complexity and importance of the role of SC nurses.
