# Supplementary material for: Workload in the Surgical Center: perceptions, activities and time spent by nurses
Source: Rev Lat Am Enfermagem. 2025 Mar 31;33:e4512. doi: 10.1590/1518-8345.7549.4512 (PMC11960620; doi:10.1590/1518-8345.7549.4512)
Supplement: Translation

# Translation: Carga de trabalho em Centro Cirúrgico: percepções, atividades e tempo despendido pelos enfermeiros assistenciais*

**DOI:** 10.1590/1518-8345.7549.4513

**Authors:** Denilse Damasceno Trevilato, Fabiana Zerbieri Martins, João Lucas Campos de Oliveira, Rita Catalina Aquino Caregnato, Tarcísio Abreu Saurin, Ana Maria Müller de Magalhães

**Affiliations:** 1 Universidade Federal do Rio Grande do Sul, Porto Alegre, RS, Brasil.; 2 Hospital Moinhos de Vento, Porto Alegre, RS, Brasil.; 3 Hospital de Clínicas de Porto Alegre, Porto Alegre, RS, Brasil.; 4 Universidade Federal de Ciências da Saúde de Porto Alegre, Porto Alegre, RS, Brasil.

**Keywords:** Papel do Profissional de Enfermagem, Carga de Trabalho, Enfermagem Perioperatória, Período Intraoperatório, Competência Profissional, Centros Cirúrgicos

## Abstract

avaliar a carga de trabalho percebida pelos enfermeiros assistenciais de centro cirúrgico e o tempo despendido nas atividades realizadas, bem como compreender as atividades que influenciam a sua percepção.

estudo de métodos mistos explanatório sequencial. Etapa quantitativa desenvolvida por *survey* com 34 enfermeiros de cinco hospitais e aferição do tempo despendido por atividade nos hospitais com maior e menor percepção de carga de trabalho, durante 129 horas de observação. Sequencialmente, realizaram-se entrevistas semiestruturadas com 12 enfermeiros. Os dados quantitativos, analisados de forma descritiva e inferencial, e qualitativos submetidos à Análise de Conteúdo, foram posteriormente integrados por conexão.

houve o predomínio do sexo feminino (88,23%) e idade média 39±8,18 anos entre os enfermeiros, com correlação moderada (r=0,502) e significativa (p=0,002) entre a idade e a percepção de carga de trabalho. As atividades gerenciais despenderam mais tempo e foram identificadas como as mais influentes na percepção da carga de trabalho associada à dimensão demanda mental.

as atividades que mais repercutem na percepção da carga de trabalho englobam o gerenciamento do setor e das pessoas. A assistência direta ao paciente foi apontada como uma atividade que proporciona a satisfação no trabalho e propósito em sua atuação.

## Destaques

**(1)** Correlação moderada e significativa da percepção da carga de trabalho com a idade. **(2)** O maior percentual do tempo mensurado foi despendido em atividades gerenciais. **(3)** A demanda mental obteve maior pontuação na percepção da carga de trabalho. **(4)** O cuidado direto ao paciente é fonte significativa de satisfação profissional. **(5)** Enfermeiros expressam perda na essência do cuidado em detrimento das outras atividades.

## Introdução

O Centro Cirúrgico (CC) é uma área de alta complexidade, na qual os fluxos de informações, materiais e pessoas demandam significativo esforço de coordenação. Nesse cenário, a atuação do enfermeiro requer habilidades de liderança da equipe e comunicação, assim como a execução de diversas atividades, tais como: gestão de pessoas, materiais e da escala de cirurgias (mapa cirúrgico), bem como o cuidado direto ao paciente ^(1)^.

As atividades dos enfermeiros no CC encontram-se relacionadas, principalmente, à organização do setor e da escala cirúrgica, no sentido de alocar os procedimentos agendados de acordo com a estrutura física e a qualificação da equipe para o melhor atendimento às necessidades de cada situação ^(2)^.

A carga de trabalho de Enfermagem pode ser definida como a quantidade e o tempo despendido para realizar uma tarefa, levando-se em conta: o grau de complexidade do cuidado de diferentes pacientes; o nível de conhecimento e as habilidades requeridas das enfermeiras para atender às diferentes necessidades dos pacientes; o cuidado direto ao paciente; além do esforço físico, mental e emocional do profissional para desenvolver as atividades de Enfermagem ^(3)^. Os estudos para a determinação da carga de trabalho por meio da mensuração de tempo gasto nas atividades de Enfermagem têm sido desenvolvidos principalmente em unidades de internação ^(4 - 6)^ e Unidade de Terapia Intensiva (UTI) ^(7)^.

Contudo, a carga de trabalho também possui uma dimensão subjetiva relacionada à maneira como cada enfermeiro vivencia suas atividades e pode ser um preditor em relação à qualidade do cuidado aos pacientes ^(8)^. Uma das ferramentas que pode ser utilizada na identificação da carga de trabalho percebida é a escala *National Aeronautics and Space Administration - Task Load Index* (NASA-TLX), utilizada, inicialmente, para coletar as informações subjetivas sobre a carga de trabalho de pilotos e controladores de tráfego aéreo ^(9)^.

Em serviços de saúde, o NASA-TLX foi empregado como forma de avaliação da carga de trabalho percebida pelo enfermeiro nas Unidades de Tratamento Intensivo Neonatal ^(8 , 10)^, adulto ^(11 - 13)^, pediátrico ^(8)^, unidade de internação pediátrica ^(14)^ e emergência ^(15 - 16)^. No CC, encontraram-se dois estudos: um que comparou as implicações de um novo projeto da mesa de instrumentação cirúrgica ^(17)^, e outro ^(18)^ que avaliou a repercussão do nível de ruído da sala cirúrgica. Como limitações, esses estudos não consideraram o conjunto de atividades realizadas pela equipe de Enfermagem, e não forneceram uma avaliação sistêmica sobre a carga de trabalho no CC apoiada em múltiplas fontes de evidências, qualitativas e quantitativas, como neste estudo de método misto proposto.

Essas considerações e a especificidade do trabalho no CC demonstraram a necessidade de empenhar os esforços sensíveis para captar a carga de trabalho percebida pelos enfermeiros deste setor e as atividades realizadas por ele. Diante disso, este estudo teve como objetivos: avaliar a carga de trabalho percebida pelos enfermeiros assistenciais do CC e o tempo despendido nas atividades realizadas, bem como compreender as atividades que influenciam a sua percepção.

## Método

### Tipo de estudo

Trata-se de um estudo de métodos mistos explanatório sequencial, em três etapas, com maior atribuição de peso à abordagem quantitativa (QUAN➝ qual) ^(19)^. Os delineamentos de cada etapa foram os seguintes: 1) *survey*; 2) estudo observacional; e 3) estudo exploratório. Nas duas primeiras etapas quantitativas (QUAN), foram identificados os pontos a serem explorados na terceira (qual). O método misto foi escolhido pela possibilidade de integração dos dados qualitativos com os quantitativos, tendo em vista complementar e enriquecer a compreensão do fenômeno em estudo, na busca de esclarecimento das atividades que influenciam a percepção de carga de trabalho pelo enfermeiro no CC.

Para a condução e o relatório desta pesquisa, foram utilizados os guias *Strengthening the Reporting Observational studies in Epidemiology* (STROBE) ^(20)^, *Consolidated criteria for Reporting Qualitative research* (COREQ) ^(21)^ e *Mixed Methods Appraisal Tool* (MMAT) ^(22)^ que buscam a transparência e a reprodutibilidade da investigação.

### Local do estudo

Realizado no CC de cinco hospitais de grande porte, públicos e privados de Porto Alegre, Rio Grande do Sul, Brasil.

### Período

Entre os meses de março e setembro de 2023, de forma sequencial, conforme as etapas previstas.

### População

Enfermeiros que desenvolviam as atividades assistenciais no CC das cinco instituições, no período de coleta, totalizando 60 enfermeiros.

### Critérios de seleção

Critérios de inclusão: enfermeiro assistencial atuante no CC com oito ou mais salas cirúrgicas e com período mínimo de seis meses de atuação no CC.

Critério de exclusão: enfermeiros gestores ou em afastamento por quaisquer motivos durante a coleta de dados.

### Definição dos participantes

Na primeira etapa (*survey*), foi utilizada uma amostra não probabilística por conveniência, composta pelo retorno de 34 (56,67%) questionários preenchidos. Para a segunda etapa observacional foram selecionados os hospitais com a maior (Hospital B) e menor (Hospital D) pontuação de percepção de carga de trabalho pelos enfermeiros que responderam ao *survey*. Foram observados 15 turnos de trabalho de seis horas em cada um dos dois hospitais, com o acompanhamento de um enfermeiro/observador/turno, totalizando 129h22min, com um total de 30 turnos e 15 enfermeiros observados. Na terceira etapa, exploratória, foram entrevistados 12 enfermeiros que trabalhavam nos dois hospitais selecionados para a etapa observacional, de acordo com o interesse e a disponibilidade, sendo a delimitação amostral obtida pela saturação dos dados ^(23)^.

### Variáveis do estudo

As variáveis foram: pontuação da percepção da carga de trabalho pela escala NASA-TLX, atividades desenvolvidas pelos enfermeiros, tempo despendido nessas atividades, variáveis sociodemográficas e do ambiente de trabalho.

### Instrumentos utilizados para a coleta das informações

Na primeira etapa foi utilizado um formulário *online* via plataforma *Research Eletronic Data Capture* (REDCap^®^) ^(24)^ contendo: Termo de Consentimento Livre e Esclarecido (TCLE); extração dos dados sociodemográficos e do ambiente de trabalho (idade, sexo, turno de trabalho, carga horária mensal de trabalho, número de salas cirúrgicas, número de enfermeiros no turno); e a escala NASA-TLX ^(9)^, instrumento traduzido e validado para o português ^(25)^.

Na escala NASA-TLX, as seis dimensões (Demanda Mental, Demanda Física, Demanda Temporal, Desempenho, Esforço e Nível de Frustração), pareadas entre si, resultaram em 15 combinações (por exemplo: Demanda Mental X Demanda Física), sendo escolhido em cada um dos pares o que mais contribuiu para a realização do trabalho. Após, as seis dimensões, separadamente, são pontuadas em uma escala contínua, em que a contagem varia de um a 100, representando o nível de influência sobre a carga de trabalho ^(9 , 22)^.

A carga de trabalho percebida foi calculada individualmente utilizando a escala NASA-TLX, seguindo quatro passos descritos por Terra ^(26)^. Primeiramente, contabilizou-se quantas vezes cada item foi considerado o mais influente entre as seis dimensões (como, por exemplo, em quantas das 15 comparações o esforço foi preponderante). Em seguida, para calcular o peso de cada dimensão, dividiu-se o número de vezes que o item foi escolhido por 15, que é o número máximo de pareamentos. No segundo passo, a pontuação da dimensão foi obtida multiplicando-se o peso da dimensão pelo nível de influência, que varia de um (pouca influência) a 100 (muita influência), com a exceção da dimensão desempenho, cujo valor foi invertido, já que quanto maior a influência, melhor. No terceiro passo, os valores foram reescalonados para que as pontuações de cada dimensão fossem parametrizadas de zero a 10, dividindo-se o valor obtido pela carga de trabalho pelo valor máximo possível (5/15=0,33), e esse resultado, por 10. Por fim, o índice global da carga de trabalho de cada indivíduo foi obtido pelo somatório das seis dimensões, também reescalonado de zero a 10. Atualmente, não existem evidências sólidas sobre os valores de referência e as classificações dos escores, dessa forma, considera-se que quanto maior for a pontuação, maior é a percepção da carga de trabalho.

Para a medida do tempo das atividades, foi utilizado o *software TrackingTime*® ^(27)^, com listagem inicial de 23 atividades executadas pelo enfermeiro do CC no cenário brasileiro, identificadas em uma *scoping review* ^(28)^. Durante a observação, foram acrescidas dez atividades, e após o reagrupamento de acordo com a frequência e a similaridade dos processos de trabalho, foi obtido resultado de 26 itens observados. Na coleta dos dados, a ativação do cronômetro ocorria no início da atividade, e a desativação, na sua conclusão, sendo pausado e reiniciado a cada interrupção.

As questões abordadas nas entrevistas da terceira etapa foram conduzidas para explorar os resultados da primeira e da segunda etapa por meio de um roteiro estruturado, com perguntas relacionadas às atividades que mais impactam a percepção da carga de trabalho, as atividades reconhecidas como importantes, mas que não conseguem realizar no seu turno de trabalho, além das atividades que trazem mais satisfação no trabalho.

### Coleta de dados

As etapas quantitativas ocorreram entre os meses de março e agosto de 2023, conduzidas por convite enviado via *e-mail* contendo o *link* do formulário e as orientações de preenchimento do instrumento. Após a análise dos resultados desta etapa foram definidos os dois CC para a etapa observatória. A equipe de coleta desta etapa foi composta pelo pesquisador principal do estudo, uma bolsista de iniciação científica e dois enfermeiros voluntários do grupo de pesquisa. Foi elaborado um tutorial sobre o uso do aplicativo *TrackingTime*® ^(27)^, que foi previamente testado pelos pesquisadores para a observação, porém, sem a realização de um teste-piloto com os enfermeiros no cenário real de prática. No início do turno de trabalho, os enfermeiros foram convidados a serem observados durante a execução de suas atividades, ao longo de todo o período de seis horas que compreendia o turno no CC.

A terceira etapa ocorreu entre os meses de agosto e setembro de 2023, conduzida pelo pesquisador principal, os participantes foram denominados com a letra “P” seguido de um numeral. As entrevistas foram gravadas, transcritas e devolvidas por *e-mail* para a validação dos respondentes, com o prazo de manifestação de uma semana. Dos participantes, uma pessoa retornou validando integralmente a transcrição, outra solicitou os ajustes, e os demais não se manifestaram dentro do prazo estabelecido. Esse processo de devolução das transcrições permitiu à equipe de pesquisadores refletir sobre a importância de envolver os respondentes na validação das informações, mesmo que nem todos retornassem, destacando-se como um ponto positivo na busca pelo rigor e a transparência da pesquisa.

### Tratamento e análise dos dados

Os dados quantitativos foram organizados em uma planilha *Google*® ^(29)^ e importados para o *softwareStatistical Package for the Social Sciences* (SPSS) ^(30)^, versão 25.0, sendo analisados de forma descritiva e analítica. A carga de trabalho percebida foi mensurada por meio da escala NASA-TLX e foi calculada individualmente de acordo com os quatro passos descritos em estudo anterior ^(26)^, com o reescalonamento da pontuação final para uma escala de um a 10.

Para a avaliação da normalidade de distribuição dos dados numéricos foram realizados os testes de Shapiro-Wilk (S-W). O *teste t de Student*, ANOVA e a correlação bivariada de Pearson foram utilizados para relacionar as variáveis sociodemográficas com a pontuação da escala NASA-TLX, adotando-se o nível de significância de 5% (p≤0,05).

A frequência e os tempos médios despendidos nas atividades foram classificadas de acordo com as três grandes áreas de atuação do enfermeiro: gestão, assistência ou ensino/pesquisa, elencadas em revisão do escopo ^(28)^. Também foi computado o tempo utilizado para o intervalo/cuidado pessoal.

Os dados qualitativos foram submetidos à Análise de Conteúdo de Bardin ^(31)^, respeitaram-se as etapas de pré-análise, exploração do material, tratamento dos resultados e a interpretação. Utilizou-se o *software NVivo*® ^(31)^, versão 14.23.2, como ferramenta auxiliar no processo de organização e sistematização dos dados. No total, foram obtidas 9h23min de gravação, resultando em 39 páginas de transcrição com cerca de 20.300 palavras. Para alcançar os consensos durante as etapas de codificação e categorização dos resultados, houve reuniões entre os pesquisadores, assegurando a consistência na interpretação dos dados.

Inicialmente, as transcrições das entrevistas foram importadas para o *software NVivo*® ^(32)^, que permite uma navegação rápida e precisa pelo *corpus*, além da possibilidade de realizar anotações diretamente no texto. Esse processo facilitou o desmembramento das unidades de registro, seu posterior agrupamento e o reagrupamento. Na etapa de categorização, foram estabelecidas as relações entre as unidades de significado identificadas na codificação, aproximando os elementos que pudessem ser combinados e/ou classificados, o que deu origem às categorias. A partir dessas categorias, novas compreensões sobre o fenômeno em estudo emergiram, enriquecendo a análise.

A integração dos dados foi realizada por conexão das três etapas, com a apresentação de metainferências a partir da análise dos resultados integrados, além da exibição conjunta em forma de *joint display*. A exibição conjunta consiste em representar visualmente, de maneira clara, os aspectos quantitativos e qualitativos de um estudo de métodos mistos. O processo iterativo de desenvolvimento dessas representações pode melhorar a compreensão da análise integrativa, ao incluir os diagramas baseados nas descobertas do estudo ^(33 - 34)^.

### Aspectos éticos

O estudo foi aprovado pelo Comitê de Ética e Pesquisa (CEP), com Certificado de Apresentação de Apreciação Ética (CAAE): 62257722.8.0000.5347.

## Resultados

Entre os 34 enfermeiros que retornaram o *survey*, verificou-se que quatro (12%) pertenciam a cada um dos Hospitais A, C e E, 10 (29%) do Hospital B e 12 (35%) do Hospital D. O predomínio da amostra foi do sexo feminino (n=30; 88,23%); com idade média de 39±8,18 anos; 12 (35,29%) trabalhavam no turno da tarde; e 24 (70,58%) realizavam carga horária mensal de 180 horas de trabalho.

O número de salas operatórias nos CC variou entre 12 e 18, com média de 15±2,83 salas. Havia, em média, três ±1,31 enfermeiros em cada turno de trabalho nos CC de cada hospital, e mediana de 24 (12;36) técnicos de Enfermagem sob sua supervisão. O número de enfermeiros por turno teve uma variação entre um e cinco, e o de técnicos, de cinco a 60.

A média do índice global da escala NASA- TLX variou de 6,05±1,00 a 7,34±1,00, sem diferença significativa entre os hospitais (Tabela 1).

**Tabela 1 -:** Pontuação das dimensões e índice global da escala NASA-TLX, conforme percepção dos enfermeiros de Centro Cirúrgico (n = 34). Porto Alegre, RS, Brasil, 2023

**Hospital**	DM*	**DF** ^†^	**DT** ^‡^	**DE** ^§^	**ES** ^||^	**FR** ^¶^	Índice global ^‡‡^
	m** (dp) ^††^	**m** ^**^ **(dp)** ^††^	m** (dp) ^††^	m** (dp) ^††^	**m** ^**^ **(dp)** ^††^	m** (dp) ^††^	**m** ^**^ **(dp)** ^††^
A	6,00 (2,40)	0,81 (1,62)	3,00 (2,54)	0,91 (0,48)	4,61 (2,02)	7,99 (2,17)	7,00 (1,22)
B	5,40 (2,22)	0,55 (1,17)	3,96 (2,3)	2,14 (1,85)	5,70 (3,48)	6,69 (3,02)	7,34 (1,00)
C	4,10 (2,64)	2,30 (3,83)	4,92 (2,42)	1,32 (1,01)	5,71 (3,75)	4,60 (3,72)	6,90 (1,32)
D	5,41 (2,03)	1,39 (1,30)	4,77 (2,86)	1,15 (1,27)	4,68 (2,70)	3,10 (2,95)	6,15 (1,08)
E	5,49 (2,55)	1,05 (0,71)	2,86 (1,63)	1,48 (1,33)	6,64 (2,40)	2,70 (4,88)	6,05 (1,00)
Total	5,33 (2,17)	1,14 (1,69)	4,12 (2,47)	1,47 (1,40)	5,32 (2,89)	4,86 (3,62)	6,68 (1,16)

*DM = Demanda Mental; ^†^DF = Demanda Física; ^‡^DT = Demanda Temporal; ^§^DE = Desempenho; ^||^ES = Esforço; ^¶^FR = Nível de Frustração; **m = Média; ^††^DP = Desvio-Padrão; ^‡‡^ANOVA (p=0,111)

Não houve correlação significativa das seguintes variáveis com o índice global da escala NASA-TLX: sexo (p=0,789), turno de trabalho (p=0,057), carga horária mensal (p=0,095), número de enfermeiros no turno (p=0,318), número de salas (p=0,080), número de salas cirúrgicas sob sua responsabilidade (p=0,564), bem como o número de técnicos de Enfermagem sob sua supervisão direta (0,245). A idade apresentou correlação moderada (r=0,502) e significativa (p=0,002) com o índice global da escala NASA-TLX (Figura 1).

**Figura 1:** - Índice global da escala NASA-TLX* de acordo com a idade (n = 34). Porto Alegre, RS, Brasil, 2023

*NASA-TLX = *National Aeronautics and Space Administration - Task Load Index*

A mensuração das atividades realizadas nos dois hospitais selecionados resultou em um total de 129h22min de observação, sendo 60h16min no Hospital B e 69h6min no Hospital D. Houve predomínio de tempo dedicado à gestão no Hospital D (p<0,001) e à assistência no Hospital B (p=0,004), com uma diferença significativa entre eles (Tabela 2).

**Tabela 2 -:** Distribuição do tempo de acordo com as atividades desenvolvidas pelos enfermeiros de Centro Cirúrgico (n = 15). Porto Alegre, RS, Brasil, 2023

**Atividade**	T* HB ^†^ **h** ^§^ **/m** ^||^ **(%)**	T* HD ^‡^ **h** ^§^ **/m** ^||^ **(%)**	T* total h ^§^ **/m** ^||^ **(%)**	**p-valor** ^¶^
**Gerenciais**	25:49 (42,84)	44:45 (64,76)	70:34 (54,55)	**<0,001**
A1. Planejamento das escalas do outro dia/turno	05:42 (9,46)	06:05 (8,80)	11:47 (9,11)	
A2. Resolução de questões administrativas	02:56 (4,87)	07:52 (11,38)	10:48 (8,35)	
A3. Gestão da equipe (dimensionamento, folgas, férias)	00:53 (1,47)	07:16 (10,52)	08:09 (6,30)	
A4. Gerenciamento da escala (encaixes, urgência, remanejo)	02:10 (3,60)	05:28 (7,91)	07:38 (5,90)	
A5. Passagem de plantão	05:18 (8,79)	02:14 (3,23)	07:32 (5,82)	
A6. Organização do CC**/supervisão das salas	01:33 (2,57)	05:39 (8,18)	07:12 (5,57)	
A7. Supervisão da equipe de Enfermagem	00:31 (0,86)	02:44 (3,96)	03:15 (2,51)	
A8. Instrumental (falta ou falha)	02:06 (3,84)	00:47 (1,13)	02:53 (2,23)	
A9. Auxílio na limpeza/montagem de sala	00:05 (0,14)	02:36 (3,76)	02:41 (2,07)	
A10. Equipamento (falha ou falta)	01:31 (2,52)	00:47 (1,13)	02:18 (1,78)	
A11. Bate mapa/ *round*	01:23 (2,30)	00:00 (0,00)	01:23 (1,07)	
A12. Gerenciamento de insumos	00:09 (0,25)	00:47 (1,13)	00:56 (0,72)	
A13. Resolução de problemas de informática	00:00 (0,00)	00:21 (0,51)	00:21 (0,27)	
A.14. Aviso anestesista	00:09 (0,25)	00:08 (0,19)	00:17 (0,22)	
A15. Registro de ocorrências e incidentes	00:06 (0,17)	00:06 (0,14)	00:12 (0,15)	
A16. Atualização da escala do outro dia para a Central de Material e Esterilização	00:08 (0,22)	00:00 (0,00)	00:08 (0,10)	
A17. Aviso/sobreaviso do transplante	00:07 (0,19)	00:00 (0,00)	00:07 (0,09)	
**Assistenciais**	30:32 (50,66)	17:17 (25:01)	47:49 (36,96)	**0,004**
A18. Avaliação do paciente e documentação da assistência prestada (processo de Enfermagem)	17:33 (29,12)	04:01 (5,81)	21:34 (16,67)	
A19. Acompanhamento de indução anestésica e auxílio da intubação difícil	06:09 (10,2)	02:32 (3,67)	08:41 (6,71)	
A20. Cuidado direto do paciente em sala cirúrgica	02:19 (3,84)	04:23 (6,34)	06:42 (5,18)	
A21. Posicionamento cirúrgico	03:20 (5,53)	02:33 (3,69)	05:53 (4,55)	
A22. Procedimentos de Enfermagem (punção, sondagem)	01:09 (1,91)	03:18 (4,78)	04:27 (3,44)	
A23. Transferência de cuidado (Centro de Tratamento Intensivo/Unidade)	01:04 (1,77)	01:55 (2,77)	02:59 (2,31)	
A24. Atendimento à intercorrência	00:00 (0,00)	00:30 (0,72)	00:30 (0,39)	
**Ensino/pesquisa**	00:04 (0,11)	00:47 (1,13)	00:51 (0,66)	1,00
A25. Capacitação da equipe	00:04 (0,11)	00:47 (1,13)	00:51 (0,66)	
**Cuidado pessoal**	03:51 (6,39)	06:17 (9,09)	10:08 (7,83)	0,024
A26. Intervalo pessoal	03:51 (6,39)	06:17 (9,09)	10:08 (7,83)	

*T = Tempo em horas e minutos; ^†^HB = Hospital B; ^‡^HD = Hospital D; ^§^h = Horas; ^||^min = Minutos; ^¶^ Mann-Whitney U test; **CC = Centro Cirúrgico

As cinco atividades mais prevalentes nos hospitais observados corresponderam a 47,14% do tempo mensurado, foram: avaliação do paciente e documentação da assistência prestada (processo de Enfermagem), planejamento das escalas do outro dia/turno, resoluções de questões administrativas, acompanhamento de indução anestésica e gestão da equipe. As 10 atividades menos frequentes totalizaram 5,45% do tempo total mensurado: equipamento (falha ou falta), bate mapa/*round,* gerenciamento de insumos, resolução de problemas de informática, aviso do anestesista, registro de ocorrências e incidentes, atualização da escala do outro dia para o centro de materiais e de esterilização, aviso/sobreaviso do transplante, atendimento à intercorrência e capacitação da equipe. O tempo utilizado para o intervalo pessoal correspondeu a 7,83% da jornada de trabalho dos enfermeiros.

A partir da análise das entrevistas em relação às atividades que mais influenciaram na percepção da carga de trabalho, emergiram seis categorias, representadas por cores diferentes, e 15 subcategorias, conforme representado na Figura 2. As categorias descritas a seguir são ilustradas com excertos extraídos das entrevistas.

**Figura 2 -:** Fatores que influenciam a percepção da carga de trabalho pelos enfermeiros de Centro Cirúrgico (n = 12). Porto Alegre, RS, Brasil, 2023

### Atividades que impactam na percepção da carga de trabalho

Os enfermeiros associam as atividades gerenciais ao aumento da percepção da carga de trabalho, principalmente em relação ao gerenciamento da escala cirúrgica, pois *[...] mesmo para escalar uma sala cirúrgica, não é simplesmente colocar a sala ali, tenho que pensar na equipe que vai estar ali, em quem vai atender, se vai ter equipamento, se vai ter material, e tudo isso demanda muito planejamento [...] (P2)* e dos profissionais de Enfermagem, que envolve o remanejo dos trabalhadores e a organização do setor para atender à demanda do CC, *[...] organizar a escala diária, não de cirurgias, mas, enfim, de pessoas, [...] tenho que lembrar que alguém está de atestado, alguém não vai vir. Fora olhar uma escala, eu tenho que olhar várias escalas para produzir uma [...] (P10).*

A sobrecarga mental é impactada pelo gerenciamento de conflitos, *gerenciar as situações, com todas essas equipes. A equipe cirúrgica, a [equipe] anestésica, com a minha equipe de Enfermagem, e com o cuidado do paciente [...] (P2)*, pode ocasionar frustração e desgaste no relacionamento entre as equipes, *[...] da gente se sentir não valorizado, se sentir menosprezado, rebaixado, várias coisas negativas em detrimento de um conflito que tu não consegues gerir (P3).*

A impossibilidade de estar presente no início de todos os procedimentos cirúrgicos e as intercorrências sem sucesso no atendimento foram atividades assistenciais identificadas que influenciaram a percepção da carga de trabalho, *[...] talvez seja pelo fato de ter tido todo esse processo de Enfermagem, que a gente realmente tem que fazer toda essa sistematização, a gente tem a assistência em sala [...] posicionar, de ajudar na indução, enfim, de organizar os equipamentos cirúrgicos, materiais cirúrgicos, [...] (P2), [...] o mais frustrante é tu estar com quatro salas e, quando vai ver [...] já entraram e tu não conseguiu acompanhar o início (P5), e [...] quando a gente não consegue reverter uma parada cardíaca, por exemplo, quando atende um bebê e [...] não tem sucesso (P2).*

A centralização das informações e atribuições na Figura**¿fig:dbo?** enfermeiro foram destacadas pelos participantes, pois *[...] parece que é tudo culpa nossa, porque a anestesia não tá funcionando, porque a sala foi bloqueada, porque o material não desceu do CME [Central de Material e Esterilização], vem tudo nas costas do enfermeiro, e aí, para o técnico, parece que os fluxos, nada funciona, que não sabemos o que está acontecendo (P5).*

### Atividades que geram satisfação

O cuidado direto ao paciente é mencionado como maior fator de satisfação, *[...] quando eu cuido do paciente direto dentro de sala [...], olhar para ele, segurar na mão, posicionar depois de anestesiado com todo o primor (P3)*, e participar de transplante, *[...] uma cirurgia dessa complexidade e com um impacto tão grande para uma família inteira, que é retirar um pedaço do fígado do pai [e implantar] na criança. (P3).*

O reconhecimento da competência e a importância da presença do enfermeiro dentro na sala cirúrgica: *[...] acho ótimo quando alguém fala: “Que bom que a enfermeira está na sala”. Porque tu sente que as pessoas estão felizes de tu estar ali, se precisarem sabem que tu vai conseguir ajudar (P3)*, e o relacionamento interpessoal com a equipe de técnicos de Enfermagem foi apontado como atividades que geram satisfação, por meio de *[...] um retorno do que eles me solicitaram. Nem que seja uma folga. Às vezes, eu consegui dar o retorno no tempo hábil para aquele técnico, não dar a resposta positiva da folga, às vezes até negativa (P4).*

### Atividades que não conseguem realizar

Ao mesmo tempo que a relação interpessoal com a equipe traz satisfação, também é reconhecida como uma atividade com tempo insuficiente para se dedicar: *[...] dar uma atenção maior para o lado pessoal da sua equipe de Enfermagem [...] é uma coisa que eu sinto falta. (P1) Poder conversar mais com os técnicos de Enfermagem de coisas triviais da vida. Conhecer melhor a história deles (P3).*

As capacitações e o acompanhamento inicial do técnico de Enfermagem ficam comprometidos diante da demanda de trabalho, *[...] uma capacitação, acompanhamento de um funcionário novo, super falho, a gente o deixa meio que largado, vai pegando sozinho [...] (P9)*, pode prejudicar o desenvolvimento e a avaliação do funcionário ao longo do ano. A necessidade de aprimorar o trabalho em equipe, aliada ao cuidado com o outro, é identificada de forma veemente, por vezes *[...] não enxergando as pessoas como pessoas por falta de tempo, [...] isso não é uma prioridade porque a gente tem outras demandas (P12)*, sendo um fator importante na busca do estabelecimento de vínculos de confiança e de qualidade.

Devido à dinâmica do CC, muitas vezes o enfermeiro não consegue se envolver no cuidado direto no início do procedimento, *[...] estar junto na hora da intubação do paciente, do posicionamento, então quando tu entra na sala e já começou a cirurgia (P5)*, o que pode ser um fator de frustração, pois, essa também foi apontada como uma das atividade que mais gera satisfação.

### Dinâmica do trabalho no CC não representa desgaste físico

Os enfermeiros não reconhecem a exigência da demanda física como influente na percepção da carga de trabalho, sendo mais frequente no atendimento às cirurgias de grande porte e complexas, como transplante, *[...] eu não tenho sensação de sair cansada fisicamente, a não ser quando eu tenho que atender um transplante e fico dentro da sala exclusivamente. Nessa situação específica, eu saio cansada fisicamente, mas me sentindo tão realizada [...] (P3).*

O desgaste físico é apontado na condição de assumir várias salas cirúrgicas, quando há diminuição do quadro de pessoal, como manifestado *[...] está com menos enfermeiros, a [...] demanda física é bem maior também (P4)*. Outro participante afirmou que, por mais que o CC represente *uma área muito grande aqui, [...] não precisa estar se deslocando para outros lugares, [por ter] uma área física delimitada […] os procedimentos de Enfermagem [...] não são [atividades] cansativas (P6).*

### A dualidade entre o tempo de experiência em CC e a percepção da carga de trabalho

A experiência pode contribuir para a maior percepção da carga de trabalho por ter uma melhor visão das necessidades do setor e da instituição, um maior envolvimento em processos e comissões, devido à qualificação e ao reconhecimento pela equipe como referência na resolução de determinadas situações, conforme expresso nos seguintes excertos: *[...] as pessoas que estão a mais tempo acabam sendo referência, não digo especialidades, mas para algumas funções (P1) [...] quanto mais experiência a gente tem, vamos tendo mais responsabilidades, [...] eu percebo que não só a idade, mas o tempo de casa, a contribuição dentro do setor (P5).*

Em contrapartida, alguns profissionais mais experientes relataram menor percepção da carga de trabalho, pois *com o passar dos anos, vai aprendendo, [...] não deixar isso influenciar tanto no trabalho (P1)*, por já ter atingido um *nível de relacionamento com a equipe cirúrgica, não digo de intimidade, mas de confiança, de credibilidade do trabalho com eles (P3).*

A inexperiência foi apontada, tanto como um fator que pode contribuir para a menor percepção da carga de trabalho pela disposição física, *porque eles têm energia e pique (P11),* e *alta capacidade de adaptação às situações (P12)*, ou por não conhecerem as rotinas, os problemas e não preverem as ocorrências evitáveis, quanto como um fator que pode aumentar essa percepção por *não saber lidar com o imprevisto ou [...] não ter expertise suficiente para lidar com as intercorrências (P2)*.

### Fatores organizacionais e individuais que impactam a percepção da carga de trabalho

O nível de envolvimento pessoal bem como o não envolvimento de colegas aumenta o sentimento de sobrecarga de trabalho, *[...] porque, se é uma pessoa que sabe ocupar o seu lugar, que sabe a diferença que faz, vai se envolver em mais frentes de trabalho (P3).*

O acúmulo de atividades gerenciais e administrativas acarreta o desvio da função assistencial do enfermeiro, pois assume demandas que poderiam ser realizadas por uma equipe administrativa: *[...] estamos perdendo aquilo que era o essencial da assistência para fazer as outras coisas, então, nossa carga está aumentando em razão disso. Os fluxos, os processos que não estão bem alinhados; normalmente, sobram para o enfermeiro. [...] o enfermeiro também não sabe dizer não, e tu não vai deixar o paciente esperando, vai receber um material quando não é para receber, vai na farmácia buscar uma coisa [...], vai atrás de uma nota fiscal de uma outra cirurgia que ninguém viu […] (P5).*

O gerenciamento da equipe e a adequação do quadro de pessoal foi levantado como um fator que contribui para o aumento da percepção de carga de trabalho em um dos hospitais, conforme o relato: *[...] a falta, dos atestados dos funcionários, do dimensionamento, da demanda do enfermeiro, da sala de urgência, de ter cirurgias mais complexas, e a gente não ter o aumento do quadro funcional (P7)*. Por outro lado, no outro hospital foi relatado que a completude do quadro dos enfermeiros contribui para uma menor percepção da carga de trabalho, pois *[...] o dimensionamento de pessoal melhorou bastante (P8) [...] porque, por mais que tenha bastante coisas para fazer, hoje, temos mais enfermeiros. Quando eu ficava sozinha, achava que a minha carga de trabalho era muito maior, porque tinha que fazer férias, a escala mensal, tinha que fazer tudo [...] e hoje sou responsável por uma escala, ainda não consigo me acostumar com isso (P10).*

A falta de reconhecimento manifestada por enfermeiros quando discorreram que ao tornar-se apenas mais um número pode contribuir para invisibilização dos esforços dedicados por eles, pois *[...] se tu se torna mais um denominador comum assim, e às vezes pela alta demanda, tu não consegue às vezes estar tão aparente a ponto de tu ter um reconhecimento. Então essa falta de reconhecimento às vezes gera uma frustração (P12).*

A exibição conjunta e a integração dos dados foram apresentadas em *joint display* seguindo a proposta de conexão, onde os achados qualitativos buscam explicar e complementar os dados quantitativos (Figura 3).

**Figura 3 -:** Integração dos dados do índice global da escala NASA-TLX, tempo despendido na realização das atividades e a percepção da carga de trabalho por enfermeiros do Centro Cirúrgico. Porto Alegre, RS, Brasil, 2023

*QUAN = Quantitativo; ^†^qual = Qualitativo; ^‡^NASA-TLX = *National Aeronautics and Space Administration - Task Load Index*; ^§^m = Média; ^||^DP = Desvio-Padrão; ^¶^h = Horas; **min = Minutos

## Discussão

A demanda mental foi a dimensão que obteve a maior pontuação na escala NASA-TLX, refletiu o quanto os enfermeiros perceberam essa dimensão como a mais desafiadora. As razões pelas quais os enfermeiros perceberam a influência dessa dimensão na carga de trabalho tornarem-se mais claras com os dados das entrevistas que relataram entre as atividades mais influentes: planejamento das escalas de pessoal e de fluxo da escala cirúrgica, gerenciamento do setor e a realização da avaliação do paciente e sua documentação, essas atividades também foram as que exigiram a maior parte do tempo desses profissionais. Esses achados convergiram com outros estudos, os quais apontaram que o papel gerencial exercido pelos enfermeiros no CC concentra-se, principalmente, na organização do setor e na dinâmica dos fluxos de trabalho ^(2 , 28 , 35)^.

Nos CC observados, as atividades não programadas estavam relacionadas, principalmente, aos remanejos de sala devido aos atrasos e/ou cancelamentos cirúrgicos, remanejo de funcionários para adequação das salas e o bloqueio para os atendimentos de urgências e transplantes. A imprevisibilidade e a dificuldade em se manter o planejamento, bem como o acúmulo de múltiplas e concomitantes atividades centradas no profissional enfermeiro, podem sobrecarregar esses trabalhadores ^(35 - 37)^. Um estudo conduzido em unidades de internação destacou que as atividades não programadas são preditoras de carga de trabalho mental ^(38)^, o que convergiu com as percepções dos enfermeiros, refletidas nas características dinâmicas do CC.

A prevalência de tempo despendido na avaliação do paciente e na documentação (16,67%) foi inferior ao resultado encontrado em um estudo norte-americano, no qual os enfermeiros despenderam 27% do tempo de atividades em registros de saúde ^(39)^. A percepção de que essa atividade impacta na demanda mental convergiu com os resultados de estudos da Holanda ^(40)^ e Itália ^(41)^, nos quais mais da metade dos enfermeiros apontaram a documentação como uma das causas de percepção de carga de trabalho elevada, o que pode ser associado à pressão e ao aumento substancial do tempo gasto nessa atividade ^(38 - 39)^. Para amenizar o impacto na carga de trabalho, é apontado que o processo de documentação deve ser realizado de forma mais integrada, com equipamentos próximos aos pacientes, isso contribui para que o registro ocorra em momento praticamente concomitante ao atendimento ^(39)^, o que pode atenuar do viés de memória, e, consequentemente, diminuir a pressão dos enfermeiros à assertividade dos seus registros.

A dimensão com menor pontuação na escala NASA-TLX foi a demanda física, o que indica que os enfermeiros não reconheceram a influência desta dimensão na percepção da carga de trabalho, em comparação às outras dimensões, isso sugere uma capacidade de adaptar-se às exigências físicas do cotidiano no CC. Os resultados de baixo escore na escala NASA-TLX e em um menor percentual (15,66%) do tempo despendido em atividades como a organização do setor e o posicionamento cirúrgico, sugeriram que os enfermeiros se sentiam adaptados à estrutura física do setor e às tarefas que exigiam esforço físico. Este achado se contrapôs aos resultados de um estudo que ressalta as consequências da demanda física na saúde dos enfermeiros, visto que uma pesquisa anterior constatou que 77,7% dos enfermeiros de sala cirúrgica relataram os distúrbios musculoesqueléticos relacionados ao trabalho ^(42)^. Ademais, em uma revisão sistemática e metanálise, os problemas lombares foram os mais presentes em enfermeiros perioperatórios, com prevalência de 62% em 19 estudos ^(43)^.

Ao se analisar a demanda temporal em conjunto com as informações das entrevistas, depreendeu-se que os enfermeiros sentiam falta de dedicarem mais tempo à supervisão da equipe e ao estabelecimento de vínculo com ela, ou seja, acompanhar os novos técnicos de Enfermagem na instituição, revisar e padronizar das atividades de assistência em sala cirúrgica, bem como promover as capacitações para a equipe. Tais achados reforçaram a necessidade de investimento em educação permanente, mesmo frente a intensa rotina do setor, pois as atualizações e capacitações da equipe de Enfermagem são consideradas essenciais para a qualidade da assistência prestada ^(1)^.

O percentual do tempo despendido (14,58%) em atividades relacionadas à presença em sala cirúrgica, suporte emocional ao paciente e atendimento direto, convergiu com a baixa pontuação da dimensão desempenho na escala NASA-TLX, demonstrou o menor envolvimento do enfermeiro com essas atividades. Os achados qualitativos contribuíram para a compreensão desses resultados quando os enfermeiros manifestaram o desejo de estarem mais próximos ao cuidado centrado no paciente. Poucos estudos têm avançado em relação ao tempo dedicado às atividades de cuidado direto ao paciente em sala cirúrgica. Um estudo anterior, desenvolvido em um cenário brasileiro, demonstrou que os enfermeiros consideraram importante suas presenças em sala cirúrgica, mesmo em procedimentos de menor complexidade ^(44)^.

Os participantes informaram supervisionar uma média de cinco salas cirúrgicas para cada enfermeiro, e consideraram esse número adequado diante das demandas de atividades. Sabe-se que adequar a equipe de Enfermagem à demanda de trabalho é um desafio, e sua importância se relaciona à segurança do paciente, sendo que o número insuficiente de pessoal, inevitavelmente, aumenta a carga de trabalho, constitui-se um fator que pode acarretar a ocorrência de eventos adversos e influenciar negativamente a qualidade do cuidado ^(45 - 46)^. O Conselho Federal de Enfermagem ^(47)^ do Brasil recomenda a dedução de horas de Enfermagem requeridas no CC por volume de procedimentos segregados em portes, prevendo um enfermeiro para cada três salas cirúrgicas (eletivas), considerando-se, também, a necessidade de um enfermeiro exclusivo em cirurgias de urgência ou de acordo com a sua complexidade. É possível inferir que a baixa atuação do enfermeiro (ou seja, o profissional com maior nível de formação) junto ao paciente, tem relação com o número reduzido de trabalhadores dessa categoria nos turnos de trabalho. Todavia, os achados deste estudo levaram à reflexão sobre a adequação da carga de trabalho não apenas baseada em números, mas, relacionada às características da instituição quanto aos procedimentos específicos realizados (como cirurgia robótica e transplantes), bem como à formação e à experiência dos profissionais.

Dentre os fatores pessoais, a correlação positiva entre a idade e a percepção da carga de trabalho demonstra a tendência do aumento da percepção de carga de trabalho, à medida que aumenta a idade. Os dados qualitativos contribuíram para o esclarecimento desses achados, onde os profissionais mais experientes foram destacados como referência na equipe, tanto em conhecimento quanto em *expertise* para a resolução de problemas. Ao se contrapor a isso, uma revisão sistemática e metanálise de resultados com o mesmo instrumento identificou uma maior percepção da carga de trabalho entre os enfermeiros inexperientes, indicou que isso pode estar relacionado ao desconhecimento do processo de Enfermagem e à insegurança para resolver as situações adversas ^(48)^.

Os fatores organizacionais podem impactar a percepção da carga de trabalho, à despeito do pouco tempo empregado em atividades relacionadas à revisão da escala cirúrgica para o dia seguinte, a comunicação da equipe anestésica e de transplante, os quais foram apontados pelos participantes como as atividades que afastaram o enfermeiro da assistência. A identificação e o estabelecimento de tarefas administrativas que podem ser delegadas sob a supervisão de enfermeiros carecem de mais estudos, inclusive com a utilização de recursos informatizados ou inteligência artificial, como propõe um protocolo de estudo alemão com o objetivo de testar e avaliar a aceitação dos usuários (Enfermagem, equipe de serviço e os pacientes) na implantação de um sistema robótico-digital para aliviar a sobrecarga de atividades do serviço de Enfermagem ^(49)^.

Os enfermeiros frequentemente associam as funções gerenciais a um aumento na carga de trabalho percebida, especialmente quando o foco está na gestão de escalas cirúrgicas, ao invés do cuidado direto aos pacientes. Essa visão, porém, não se resume apenas à sobrecarga mental, mas parece revelar os aspectos mais profundos ligados à formação e aos valores da Enfermagem, historicamente, ao priorizar as atividades assistenciais em detrimento das habilidades gerenciais, de ensino e de pesquisa. A dicotomia entre o cuidado direto ao paciente e a gestão dos serviços reflete uma construção cultural que valoriza mais as práticas assistenciais em detrimento às posições de liderança exercidas por enfermeiros. Essa condição se revela preocupante, à medida que pode incorrer no desenvolvimento da competência gerencial do enfermeiro, e a gestão dos serviços possa ser considerada como dispensável. Esse cenário pode acarretar em consequências graves para a profissão, considerando que a própria OMS ^(50)^ ressaltou o impacto positivo da liderança de enfermeiros gestores nos serviços, mas também, sinalizou que esta presença ainda é em número restrito, e dificulta a efetivação de políticas de saúde em escala global.

Considera-se como limitação deste estudo o número de profissionais e instituições participantes, o que pode restringir a generalização dos resultados. As características específicas dos participantes e a variedade de práticas e abordagens podem não refletir a totalidade da população em questão. Dessa forma, sugere-se ampliar a diversidade e a representatividade da amostra em estudos futuros, buscando fortalecer a robustez e a aplicabilidade das conclusões obtidas.

Os resultados deste estudo representam novos *insights* de como as atividades realizadas e o tempo despendido pelos enfermeiros podem repercutir na percepção da carga de trabalho. A análise do tempo despendido em diferentes atividades tem potencial para contribuir na racionalização do tempo e dos recursos, bem como no dimensionamento adequado das equipes. A compreensão aprofundada pode oferecer subsídios na elaboração de estratégias para otimizar a distribuição de tarefas, melhorar a eficiência operacional e promover um ambiente de trabalho mais equilibrado e satisfatório para esses profissionais. O estudo pode subsidiar as discussões de entidades de classe e sociedades de especialidade sobre as atribuições do enfermeiro no CC e seu maior envolvimento no cuidado direto ao paciente, o que irá implicar na revisão da composição das equipes.

## Conclusão

Ao avaliar a carga de trabalho percebida e o tempo despendido nas atividades realizadas pelos enfermeiros do CC, constatou-se a maior pontuação atribuída na dimensão demanda mental, com predomínio de atividades relacionadas ao gerenciamento do setor e de pessoas, além das atividades relacionadas à avaliação do paciente e à documentação. Embora as tarefas gerenciais demandem parcela preponderante do tempo desses profissionais, a conexão direta com o cuidado ao paciente durante os procedimentos cirúrgicos, mesmo em menor proporção, recompensa-os proporcionando a satisfação no trabalho e o propósito em sua atuação. Essa dualidade entre a gestão eficiente e a entrega de cuidados diretos ao paciente destaca a complexidade e a importância do papel dos enfermeiros no CC.

# Translation: Carga de trabajo en Centro Quirúrgico: percepciones, actividades y tiempo utilizado por los enfermeros asistenciales*

**DOI:** 10.1590/1518-8345.7549.4511

**Authors:** Denilse Damasceno Trevilato, Fabiana Zerbieri Martins, João Lucas Campos de Oliveira, Rita Catalina Aquino Caregnato, Tarcísio Abreu Saurin, Ana Maria Müller de Magalhães

**Affiliations:** 1 Universidade Federal do Rio Grande do Sul, Porto Alegre, RS, Brasil.; 2 Hospital Moinhos de Vento, Porto Alegre, RS, Brasil.; 3 Hospital de Clínicas de Porto Alegre, Porto Alegre, RS, Brasil.; 4 Universidade Federal de Ciências da Saúde de Porto Alegre, Porto Alegre, RS, Brasil.

**Keywords:** Rol de la Enfermera, Carga de Trabajo, Enfermería Perioperatoria, Periodo Intraoperatorio, Competencia Profesional, Centros Quirúrgicos

## Abstract

evaluar la carga de trabajo percibida por los enfermeros asistenciales de centro quirúrgico y el tiempo utilizado en las actividades realizadas, así como comprender las actividades que influencian su percepción.

estudio de métodos mixtos explicativo secuencial. Etapa cuantitativa desarrollada por *survey* en 34 enfermeros de cinco hospitales y medición del tiempo utilizado por actividades en los hospitales con mayor y menor percepción de carga de trabajo, durante 129 horas de observación. Secuencialmente, se realizaron entrevistas semiestructuradas con 12 enfermeros. Los datos cuantitativos, fueron analizados de forma descriptiva e inferencial y los cualitativos después de sometidos al análisis de contenido, fueron posteriormente integrados por conexión.

hubo predominio del sexo femenino (88,23%) y la edad media fue de 39±8,18 años entre los enfermeros, con correlación moderada (r=0,502) y significación (*p*=0,002) entre edad y percepción de la carga de trabajo. Las actividades administrativas utilizaron más tiempo y fueron identificadas como las más influentes en la percepción de la carga de trabajo asociada a la dimensión demanda mental.

las actividades que más repercuten en la percepción de la carga de trabajo engloban la administración del sector y las personas. La asistencia directa al paciente fue apuntada como la actividad que proporciona satisfacción en el trabajo y propósito en la actuación.

## Destacados

**(1)** Correlación moderada y significativa de la percepción de la carga de trabajo con la edad. **(2)** El mayor porcentaje del tiempo medido fue utilizado en actividades administrativas. **(3)** La demanda mental obtuvo mayor puntuación en la percepción de la carga de trabajo. **(4)** El cuidado directo al paciente es fuente significativa de satisfacción profesional. **(5)** Los enfermeros expresan pérdida en la esencia del cuidado en detrimento a otras actividades.

## Introducción

El centro quirúrgico (CQ) es un área de alta complejidad, en la cual los flujos de informaciones, materiales y personas, demandan un esfuerzo significativo de coordinación. En ese escenario, la actuación del enfermero requiere habilidades de liderazgo del equipo y comunicación, así como la ejecución de diversas actividades, como la gestión de personas, materiales y de la escala de cirugías (mapa quirúrgico), así como el cuidado directo al paciente ^(1)^.

Las actividades de los enfermeros en el CQ se encuentran relacionadas, principalmente, con la organización del sector y con la escala quirúrgica, en el sentido de asignar los procedimientos programados de acuerdo con la estructura física y calificación del equipo, para obtener la mejor atención a las necesidades de cada situación ^(2)^.

La carga de trabajo de la enfermería puede ser definida como la cantidad y el tiempo utilizado para realizar una tarea, considerando: el grado de complejidad del cuidado de diferentes pacientes; el nivel de conocimiento y habilidades requeridas de las enfermeras, para atender diferentes necesidades de los pacientes; el cuidado directo al paciente; y además el esfuerzo físico, mental y emocional del profesional para desarrollar las actividades de enfermería ^(3)^. Los estudios para determinar la carga de trabajo, a través de la medida del tiempo utilizado en las actividades de enfermería, han sido desarrollados principalmente en Unidades de Internación ^(4 - 6)^ y Unidades de Terapia Intensiva ^(7)^.

Sin embargo, la carga de trabajo también posee una dimensión subjetiva relacionada a la manera como cada enfermero experimenta sus actividades, pudiendo ser un predictor de la calidad del cuidado con los pacientes ^(8)^. Una de las herramientas que puede ser utilizada en la identificación de la carga de trabajo es la escala *National Aeronautics and Space Administration - Task Load Index* (NASA-TLX), utilizada inicialmente para recolectar informaciones subjetivas sobre la carga de trabajo de pilotos y controladores de tráfico aéreo ^(9)^.

En los servicios de salud, el NASA-TLX fue empleado como forma de evaluación de la carga de trabajo percibida por el enfermero, en las Unidades de Tratamiento Intensivo Neonatal ^(8 , 10)^, Adulto ^(11 - 13)^, Pediátrico ^(8)^, Unidad de Internación Pediátrica ^(14)^ y Emergencia ^(15 - 16)^. Encontramos dos estudios sobre CQ: uno comparó las implicaciones de un nuevo proyecto de la mesa de instrumentación quirúrgica ^(17)^ y el otro ^(18)^ evaluó la repercusión del nivel de ruido en la sala quirúrgica. Como limitaciones, podemos mencionar que esos estudios no consideran el conjunto de actividades realizadas por el equipo de enfermería y no suministran una evaluación sistémica sobre la carga de trabajo en el CQ, apoyada en múltiples fuentes de evidencias, cualitativas y cuantitativas, como es el caso de este estudio de método mixto propuesto.

Esas consideraciones y la especificidad del trabajo en el CQ demuestran la necesidad de realizar esfuerzos sensibles para captar la carga de trabajo, percibida por los enfermeros de ese sector y las actividades realizadas por los mismos. Delante de eso, este estudio tuvo como objetivos: evaluar la carga de trabajo percibida por los enfermeros asistenciales de un centro quirúrgico y medir el tiempo utilizado en las actividades realizadas, para así poder comprender las actividades que influencian su percepción.

## Método

### Tipo de estudio

Se trata de un estudio de métodos mixtos explicativo y secuencial, realizado en tres etapas, con mayor atribución de peso al abordaje cuantitativo (CUAN➝ cual) ^(19)^. Los delineamientos de cada etapa fueron los siguientes: 1) *survey*; 2) estudio observacional; y 3) estudio exploratorio. En las dos primeras etapas cuantitativas (CUAN), fueron identificados los puntos a ser explorados en la tercera etapa (cual). El método mixto fue seleccionado por permitir integrar los datos cualitativos con los cuantitativos, con la finalidad de complementar y enriquecer la comprensión del fenómeno estudiado, tratando de esclarecer las actividades que influencian la percepción de la carga de trabajo del enfermero en el CQ.

Para la conducción y el informe de esta investigación, fueron utilizados los guías *Strengthening the Reporting Observational studies in Epidemiology* (STROBE) ^(20)^, *Consolidated criteria for Reporting Qualitative research* (COREQ) ^(21)^ y *Mixed Methods Appraisal Tool* (MMAT) ^(22)^buscando transparencia y reproductibilidad de la investigación.

### Local del estudio

Los locales del estudio del CQ fueron cinco hospitales de gran porte, públicos y privados de Porto Alegre, estado de Rio Grande del Sur, en Brasil.

### Período

Se realizó entre marzo y septiembre de 2023, de forma secuencial y conforme a las etapas previstas.

### Población

Fueron los enfermeros que desarrollaban actividades asistenciales en el CQ de las cinco instituciones, en el período de la recolecta, totalizando 60 enfermeros.

### Criterios de selección

- Criterios de inclusión: enfermeros asistenciales actuantes en un CQ, con ocho o más salas quirúrgicas y un período mínimo de seis meses de actuación en ese centro.

- Criterio de exclusión: enfermeros gestores o en licencia (por cualquier motivo) durante la recolección de datos.

### Definición de los participantes

En la primera etapa (*survey*), fue utilizada una muestra no probabilística por conveniencia, compuesta por el retorno de 34 (56,67%) cuestionarios respondidos. Para la segunda etapa observacional fueron seleccionados los hospitales con la mayor (Hospital B) y menor (Hospital D) puntuación de percepción de carga de trabajo por los enfermeros que respondieron a la *survey*. Fueron observados 15 turnos de trabajo de seis horas en cada uno de los dos hospitales, con acompañamiento de un enfermero/observador/turno, totalizando 129:22h, con un total de 30 turnos y 15 enfermeros observados. En la tercera etapa exploratoria, fueron entrevistados 12 enfermeros que trabajaban en los dos hospitales seleccionados para la etapa observacional, de acuerdo con el interés y disponibilidad, siendo la delimitación de la muestra obtenida por la saturación de los datos ^(23)^.

### Variables del estudio

Las variables fueron: la puntuación de la percepción de la carga de trabajo medida por la escala NASA-TLX; las actividades desarrolladas por los enfermeros; el tiempo utilizado en esas actividades; las variables sociodemográficas; y, las variables del ambiente de trabajo.

### Instrumentos utilizados para la recolección de las informaciones

En la primera etapa fue utilizado un formulario *online* vía plataforma *Research Eletronic Data Capture* (REDCap^®^) ^(24)^, que contenía el Término de Consentimiento Libre e Informado (TCLI); la extracción de datos sociodemográficos y del ambiente de trabajo (edad, sexo, turno de trabajo, carga horaria mensual de trabajo, número de salas quirúrgicas, número de enfermeros en el turno); y la escala NASA-TLX ^(9)^ traducida y validada para el portugués ^(25)^.

En la escala NASA-TLX, las seis dimensiones (Demanda Mental, Demanda Física, Demanda Temporal, Desempeño, Esfuerzo y Nivel de Frustración), fueron pareadas entre sí, resultando en 15 combinaciones (por ejemplo: Demanda Mental X Demanda Física). Fue seleccionado en cada uno de los pares lo que más contribuyó para la realización del trabajo. Después, las seis dimensiones, separadamente, fueron puntuadas en una escala continua, en la cual el conteo varía de 01 a 100, representando el nivel de influencia sobre la carga de trabajo ^(9 , 22)^.

La carga de trabajo percibida fue calculada - individualmente utilizando la escala NASA-TLX - siguiendo los cuatro pasos descritos por Terra ^(26)^. Primero se contabilizó cuantas veces cada ítem fue considerado el más influyente, entre las seis dimensiones (por ejemplo, en cuantas de las 15 comparaciones el esfuerzo fue preponderante). En seguida, para calcular el peso de cada dimensión, se dividió por 15 el número de veces el ítem fue escogido; este valor sería el número máximo de pareos. En el segundo paso, la puntuación de la dimensión fue obtenida multiplicando el peso de la dimensión por el nivel de influencia, que varío de 01 (poca influencia) a 100 (mucha influencia); excepto la dimensión desempeño, cuyo valor fue invertido, ya que cuanto mayor es la influencia, mejor será el desempeño. En el tercer paso, los valores fueron reescalonados para que las puntuaciones de cada dimensión fuesen parametrizadas de 0 a 10, dividiendo el valor obtenido de la carga de trabajo por el valor máximo posible (5/15=0,33); y, ese resultado dividido por 10. Por último, el índice global de la carga de trabajo de cada individuo fue obtenido por la sumatoria de las seis dimensiones, también reescalonado de 0 a 10. Actualmente, no existen evidencias sólidas sobre valores de referencia y clasificaciones de los puntajes; así, se considera que cuanto mayor es la puntuación, mayor será la percepción de la carga de trabajo.

Para la medida del tiempo de las actividades, fue utilizado el *software TrackingTime*® ^(27)^, con una lista inicial de 23 actividades ejecutadas por el enfermero del CQ, en el escenario brasileño, identificadas en una *scoping review* ^(28)^. Durante la observación, fueron adicionadas diez actividades; después de ser reagrupadas, de acuerdo con la frecuencia y similitud de los procesos de trabajo, fue obtenido el resultado final de los 26 ítems observados. En la recolección de estos datos, la activación del cronómetro sucedía en el inicio de la actividad y la desactivación se realizaba en la conclusión, siendo pausado y reiniciado a cada interrupción.

Las cuestiones abordadas en las entrevistas de la tercera etapa fueron realizadas para explorar los resultados de la primera y de la segunda etapa, a través de un guión estructurado, con preguntas relacionadas a las actividades que más impactan la percepción de la carga de trabajo, en las actividades reconocidas como importantes, pero que no consiguen realizar en su turno de trabajo, además de otras actividades que provocan más satisfacción en el trabajo.

### Recolección de datos

Las etapas cuantitativas ocurrieron entre marzo y agosto de 2023; fueron realizadas por invitación vía e-mail que contenía el *link* del formulario y las orientaciones del llenado del instrumento. Después del análisis de los resultados de esta etapa fueron definidos los dos CQ para la etapa de observación. El equipo de recolección de esta etapa estuvo compuesto por el investigador principal del estudio, una becada de iniciación científica y dos enfermeros voluntarios del grupo de investigación. Fue elaborado un tutorial sobre el uso del aplicativo *TrackingTime*® ^(27)^, que fue previamente comprobado por los investigadores para la observación; pero, sin la realización de un test piloto con enfermeros en el escenario real de la práctica. En el inicio del turno de trabajo, los enfermeros fueron invitados a ser observados durante la ejecución de sus actividades, a lo largo de todo el período de seis horas que comprendía el turno en el CQ.

La tercera etapa ocurrió entre agosto y septiembre de 2023, realizada por el investigador principal; los participantes fueron denominados con la letra “P” seguida de un número. Las entrevistas fueron grabadas, transcritas y devueltas por e-mail para la validación de los respondientes, con plazo de manifestación de una semana. Entre los participantes, una persona retornó validando integralmente la transcripción, otra solicitó ajustes y los demás no se manifestaron dentro del plazo establecido. Ese proceso de devolución de las transcripciones le permitió al equipo de investigadores reflexionar sobre la importancia de envolver a los respondientes en la validación de las informaciones, considerando que no todos responderían, destacándose como un punto positivo en lo que se refiere a la búsqueda de rigor y transparencia de la investigación.

### Tratamiento y análisis de los datos

Los datos cuantitativos fueron organizados en una planilla *Google*® ^(29)^ e importados para el *softwareStatistical Package for the Social Sciences* (SPSS) ^(30)^, versión 25.0; siendo analizados de forma descriptiva y analítica. La carga de trabajo percibida, medida a través de la escala NASA-TLX, fue calculada individualmente de acuerdo con los cuatro pasos descritos en estudio anterior ^(26)^, con reescalonamiento de la puntuación final para una escala de 1 a 10.

Para evaluación de la normalidad de la distribución de los datos numéricos, fueron realizadas las pruebas de Shapiro-Wilk (S-W). El test t de Student, ANOVA y la correlación bivariada de Pearson fueron utilizados para relacionar las variables sociodemográficas con la puntuación de la escala NASA-TLX, adoptándose el nivel de significación de 5% (p≤0,05).

La frecuencia y los tiempos medios utilizados en las actividades fueron clasificados de acuerdo con tres grandes áreas de actuación del enfermero: gestión, asistencia y enseñanza/investigación, asignadas en revisión del objetivo ^(28)^. También fue computado el tiempo utilizado para intervalo/cuidado personal.

Los datos cualitativos fueron sometidos al análisis de contenido de Bardin ^(31)^, se respetaron las etapas de preanálisis, exploración del material, tratamiento de los resultados e interpretación. Se utilizó el *software* NVivo® ^(31)^ (versión 14.23.2) como herramienta auxiliar en el proceso de organización y sistematización de los datos. En total, fueron obtenidas 9 horas y 23 minutos de grabación, resultando en 39 páginas de transcripción, con cerca de 20.300 palabras. Para alcanzar el consenso durante las etapas de codificación y categorización de los resultados, se realizaron reuniones entre los investigadores, asegurando la consistencia de la interpretación de los datos.

Inicialmente, las transcripciones de las entrevistas fueron importadas para el *software* NVivo® ^(32)^, lo que permitió una navegación rápida y precisa por el *corpus*; además permitió realizar anotaciones directamente en el texto. Ese proceso facilitó el desmembramiento de las unidades de registro, su posterior agrupamiento y reagrupamiento. En la etapa de categorización, fueron establecidas relaciones entre las unidades de significado identificadas en la codificación, aproximando elementos que pudiesen ser combinados y/o clasificados, lo que dio origen a las categorías. A partir de esas categorías, nuevas comprensiones sobre el fenómeno estudiado surgieron, enriqueciendo el análisis.

La integración de los datos fue realizada por conexión de las tres etapas, con presentación de meta-inferencias a partir del análisis de los resultados integrados; además posibilitó la exhibición conjunta en forma de *joint display*. La exhibición conjunta consiste en representar visualmente, de manera clara, los aspectos cuantitativos y cualitativos de un estudio de métodos mixtos. El proceso interactivo de desarrollo de esas representaciones puede mejorar la comprensión del análisis de integración, al incluir diagramas basados en los hallazgos del estudio ^(33 - 34)^.

### Aspectos éticos

El estudio fue aprobado por el Comité de Ética e Investigación con Certificado de Presentación de Apreciación Ética (CAAE): 62257722.8.0000.5347.

## Resultados

Entre los 34 enfermeros que retornaron el *survey*, se verificó que cuatro (12%) pertenecían a cada uno de los Hospitales A, C y E, 10 (29%) al Hospital B y 12 (35%) al Hospital D. El predominio de la muestra fue del sexo femenino (n=30; 88,23%); edad media de 39±8,18 años; 12 (35,29%) trabajaban en el turno de la tarde; y 24 (70,58%) realizaban carga horaria mensual de 180 horas de trabajo.

El número de salas de operación en los CQ varió entre 12 y 18, con una media de 15±2,83 salas. Había, en media, tres ±1,31 enfermeros en cada turno de trabajo en los CQ de cada hospital; la mediana fue de 24 (12;36) técnicos de enfermería bajo supervisión de los enfermeros. El número de enfermeros por turno varió entre uno y cinco, y el de técnicos de cinco a 60.

La media del índice global de la escala NASA- TLX varió entre 6,05±1,00 y 7,34±1,00, sin diferencia significativa entre los hospitales (Tabla 1).

**Tabla 1 -:** Puntuación de las dimensiones e índice global de la escala NASA-TLX, conforme percepción de los enfermeros del centro quirúrgico (n = 34). Porto Alegre, RS, Brasil, 2023

**Hospital**	DM*	**DF** ^†^	**DT** ^‡^	**DE** ^§^	**ES** ^||^	**FR** ^¶^	Índice global ^‡‡^
	m** (de) ^††^	**m** ^**^ **(de)** ^††^	m** (de) ^††^	m** (de) ^††^	**m** ^**^ **(de)** ^††^	m** (de) ^††^	**m** ^**^ **(de)** ^††^
A	6,00 (2,40)	0,81 (1,62)	3,00 (2,54)	0,91 (0,48)	4,61 (2,02)	7,99 (2,17)	7,00 (1,22)
B	5,40 (2,22)	0,55 (1,17)	3,96 (2,3)	2,14 (1,85)	5,70 (3,48)	6,69 (3,02)	7,34 (1,00)
C	4,10 (2,64)	2,30 (3,83)	4,92 (2,42)	1,32 (1,01)	5,71 (3,75)	4,60 (3,72)	6,90 (1,32)
D	5,41 (2,03)	1,39 (1,30)	4,77 (2,86)	1,15 (1,27)	4,68 (2,70)	3,10 (2,95)	6,15 (1,08)
E	5,49 (2,55)	1,05 (0,71)	2,86 (1,63)	1,48 (1,33)	6,64 (2,40)	2,70 (4,88)	6,05 (1,00)
Total	5,33 (2,17)	1,14 (1,69)	4,12 (2,47)	1,47 (1,40)	5,32 (2,89)	4,86 (3,62)	6,68 (1,16)

*DM = Demanda Mental; ^†^DF = Demanda Física; ^‡^DT = Demanda Temporal; ^§^DE = Desempeño; ^||^ES = Esfuerzo; ^¶^FR = Nivel de Frustración; **m = Media; ^††^de = Desviación Estándar; ^‡‡^ANOVA (p=0,111)

No hubo correlación significativa de las siguientes variables con el índice global de la escala NASA-TLX: sexo (p=0,789), turno de trabajo (p=0,057), carga horaria mensual (p=0,095), número de enfermeros en el turno (p=0,318), número de salas (p=0,080), número de salas quirúrgicas bajo responsabilidad del enfermero (p=0,564), así como el número de técnicos de enfermería bajo su supervisión directa (0,245). La edad presentó correlación moderada (r=0,502) y la significación (p=0,002) con el índice global de la escala NASA-TLX (Figura 1).

**Figura 1 -:** Índice global de la escala NASA-TLX* de acuerdo con la edad (n = 34). Porto Alegre, RS, Brasil, 2023

*NASA-TLX = *National Aeronautics and Space Administration - Task Load Index*

La medida de las actividades realizadas en los dos hospitales seleccionados resultó en un total de 129:22h de observación, siendo 60:16h en el Hospital B y 69:06h en el Hospital D. Hubo predominio de tiempo dedicado a la administración en el Hospital D (p<0,001) y a la asistencia en el Hospital B (p=0,004), con diferencia significativa entre ellos (Tabla 2).

**Tabla 2 -:** Distribución del tiempo de acuerdo con las actividades desarrolladas por los enfermeros del centro quirúrgico (n = 15). Porto Alegre, RS, Brasil, 2023

**Actividad**	T* HB ^†^ **h** ^§^ **/m** ^||^ **(%)**	T* HD ^‡^ **h** ^§^ **/m** ^||^ **(%)**	T* total h ^§^ **/m** ^||^ **(%)**	**p-valor** ^¶^
**Administrativas**	25:49 (42,84)	44:45 (64,76)	70:34 (54,55)	**<0,001**
A1. Planificación de las escalas del otro día/turno	05:42 (9,46)	06:05 (8,80)	11:47 (9,11)	
A2. Resolución de asuntos administrativos	02:56 (4,87)	07:52 (11,38)	10:48 (8,35)	
A3. Administración del equipo (dimensionamiento, descansos, vacaciones)	00:53 (1,47)	07:16 (10,52)	08:09 (6,30)	
A4. Administración de la escala (encajes, urgencia, reubicaciones)	02:10 (3,60)	05:28 (7,91)	07:38 (5,90)	
A5. Pasaje de turno	05:18 (8,79)	02:14 (3,23)	07:32 (5,82)	
A6. Organización del centro quirúrgico/supervisión de las salas	01:33 (2,57)	05:39 (8,18)	07:12 (5,57)	
A7. Supervisión del equipo de enfermería	00:31 (0,86)	02:44 (3,96)	03:15 (2,51)	
A8. Instrumental (falta o falla)	02:06 (3,84)	00:47 (1,13)	02:53 (2,23)	
A9. Auxilio en la limpieza/montaje de sala	00:05 (0,14)	02:36 (3,76)	02:41 (2,07)	
A10. Equipamiento (falla o falta)	01:31 (2,52)	00:47 (1,13)	02:18 (1,78)	
A11. Identificar factores de cancelación/ *round*	01:23 (2,30)	00:00 (0,00)	01:23 (1,07)	
A12. Gerenciamiento de insumos	00:09 (0,25)	00:47 (1,13)	00:56 (0,72)	
A13. Resolución de problemas de informática	00:00 (0,00)	00:21 (0,51)	00:21 (0,27)	
A.14. Aviso anestesista	00:09 (0,25)	00:08 (0,19)	00:17 (0,22)	
A15. Registro de ocurrencias e incidentes	00:06 (0,17)	00:06 (0,14)	00:12 (0,15)	
A16. Actualización de la escala del otro día para Central de Material y Esterilización	00:08 (0,22)	00:00 (0,00)	00:08 (0,10)	
A17. Aviso sobre-aviso de trasplante	00:07 (0,19)	00:00 (0,00)	00:07 (0,09)	
**Asistenciales**	30:32 (50,66)	17:17 (25:01)	47:49 (36,96)	**0,004**
A18. Evaluación del paciente y documentación de la asistencia prestada (proceso de enfermería)	17:33 (29,12)	04:01 (5,81)	21:34 (16,67)	
A19. Acompañamiento de inducción anestésica y auxilio de intubación difícil	06:09 (10,2)	02:32 (3,67)	08:41 (6,71)	
A20. Cuidado directo del paciente en la sala quirúrgica	02:19 (3,84)	04:23 (6,34)	06:42 (5,18)	
A21. Posicionamiento quirúrgico	03:20 (5,53)	02:33 (3,69)	05:53 (4,55)	
A22. Procedimientos de enfermería (punción, sondaje)	01:09 (1,91)	03:18 (4,78)	04:27 (3,44)	
A23. Transferencia de cuidado (Centro de Tratamiento Intensivo/Unidad)	01:04 (1,77)	01:55 (2,77)	02:59 (2,31)	
A24. Atención a intercurrencias	00:00 (0,00)	00:30 (0,72)	00:30 (0,39)	
**Enseñanza/investigación**	00:04 (0,11)	00:47 (1,13)	00:51 (0,66)	1,00
A25. Capacitación del equipo	00:04 (0,11)	00:47 (1,13)	00:51 (0,66)	
**Cuidado personal**	03:51 (6,39)	06:17 (9,09)	10:08 (7,83)	0,024
A26. Intervalo personal	03:51 (6,39)	06:17 (9,09)	10:08 (7,83)	

*T = Tiempo en horas y minutos; ^†^HB = Hospital B; ^‡^HD = Hospital D; ^§^h = Horas; ^||^min = Minutos; ^¶^ *Mann-Whitney U test*

Las cinco actividades más prevalentes, en los hospitales observados, correspondió a 47,14% del tiempo medido, las que fueron: evaluación del paciente y documentación de la asistencia prestada (proceso de enfermería); planificación de las escalas del otro día/turno; resoluciones de asuntos administrativas; acompañamiento de inducción anestésica; y, gestión del equipo. Las 10 actividades menos frecuentes totalizaron 5,45% del tiempo total medido: equipamiento (falla o falta); identificación posibles factores de cancelación de cirugías/*round;* administración de insumos; resolución de problemas de informática; aviso al anestesista; registro de ocurrencias e incidentes; actualización de la escala del otro día para el centro de materiales y esterilización; aviso sobre-aviso de trasplante; atención a intercurrencia; y, capacitación del equipo. El tiempo utilizado para intervalo personal correspondió a 7,83% de la jornada de trabajo de los enfermeros.

A partir del análisis de las entrevistas con relación a las actividades que más influenciaron en la percepción de la carga de trabajo, surgieron seis categorías, que se representan con colores diferentes, y 15 subcategorías, conforme representado en la Figura 2. Las categorías descritas a seguir son ilustradas con trechos extraídos de las entrevistas.

**Figura 2 -:** Factores que influencian la percepción de la carga de trabajo de los enfermeros del Centro Quirúrgico (n = 12). Porto Alegre, RS, Brasil, 2023

### Actividades que impactan en la percepción de la carga de trabajo

Los enfermeros asocian las actividades administrativas al aumento de la percepción de la carga de trabajo, principalmente con relación a la administración de la escala quirúrgica, ya que:

*[...] mismo para escalar una sala quirúrgica, no es simplemente colocar la sala allí, tengo que pensar en el equipo que va estar allí, en quien va atender, si va a tener equipamiento, si va a tener material, y todo eso demanda mucha planificación [...] (P2)* y de los profesionales de enfermería, que tiene que ver con nueva asignación de los trabajadores y organización del sector para atender la demanda del CQ, *[...] organizar la escala diaria, no de cirugías, pero en fin, de personas, [...] tengo que recordar que alguien está de certificado médico, alguien no va a venir. Además de mirar una escala, yo tengo que examinar varias escalas para producir una [...] (P10).*

La sobrecarga mental es impactada por la administración de conflictos, *administrar las situaciones, con todos esos equipos. El equipo quirúrgico, el [equipo] de anestesia, con mi equipo de enfermería, y con el cuidado al paciente [...] (P2)*, pudiendo ocasionar frustración y desgaste en el relacionamiento entre los equipos, *[...] las personas se sienten no valorizadas, se siente menospreciadas, rebajadas, varias cosas negativas en detrimento de un conflicto que tú no consigues administrar (P3)*.

La imposibilidad de estar presente en el inicio de todos los procedimientos quirúrgicos e intercurrencias sin éxito en el atención, fueron actividades asistenciales identificadas que influencian la percepción de la carga de trabajo, *[...] tal vez sea por el hecho de haber tenido todo ese proceso de enfermería, que nosotras realmente tenemos que hacer toda esa sistematización, nosotras tenemos la asistencia en sala [...] posicionar, ayudar en la inducción, en fin, organizar equipamientos quirúrgicos, materiales quirúrgicos, [...] (P2), [...] lo más frustrante es tú estar con cuatro salas y, cuando vas a ver [...] ya entraron y tú no consigues acompañar el inicio (P5), y [...] cuando nosotras no conseguimos reverter una parada cardíaca, por ejemplo, cuando se atiende un bebé y [...] no hay éxito (P2).*

La centralización de las informaciones y atribuciones en la figura del enfermero fueron destacadas por los participantes, ya que *[...] parece que es todo culpa nuestra, porque la anestesia no está funcionando, porque la sala fue bloqueada, porque el material no llegó del CME [Central de Material y Esterilización], cae todo en la espalda del enfermero, y ahí, para el técnico, parece que los flujos, nada funciona, que no sabemos lo que está sucediendo (P5).*

### Actividades que generan satisfacción

El cuidado directo al paciente es mencionado como el mayor factor de satisfacción, *[...] cuando yo cuido del paciente directo dentro de sala [...], mirar para él, asegurar la mano, posicionar después de anestesiado con todo el primor (P3), y* participar de trasplante, *[...] una cirugía de esa complejidad y con un impacto tan grande para una familia entera, que es retirar un pedazo del hígado del padre [e implantar] en el niño. (P3).*

El reconocimiento de la competencia e importancia de la presencia del enfermero dentro de la sala quirúrgica: *[...] creo óptimo cuando alguien dice: “Qué bueno que la enfermera está en la sala!”. Porque tú sientes que las personas están felices de tú estar allí, como si necesitaran saber que tú vas a conseguir ayudar (P3),* y el relacionamiento interpersonal con el equipo de técnicos de enfermería fue apuntado como actividades que generan satisfacción, a través de *[...] un retorno de lo que ellos me solicitaron. Ni que sea un descanso. A veces, yo conseguí dar el retorno en el tiempo hábil para aquel técnico, no dar la respuesta positiva del tiempo libre, a veces hasta negativa (P4).*

### Actividades que no consiguen realizar

Al mismo tiempo en que la relación interpersonal con el equipo trae satisfacción, también es reconocida como una actividad con tiempo insuficiente para dedicarse: *[...] dar una atención mayor para el lado personal de su equipo de enfermería [...] es una cosa que yo siento falta. (P1) Poder conversar más con los técnicos de enfermería de cosas triviales de la vida. Conocer mejor la historia de ellos (P3).*

Las capacitaciones y el acompañamiento inicial del técnico de enfermería quedan comprometidos delante de la demanda de trabajo, *[...] una capacitación, acompañamiento de un funcionario nuevo, que falla mucho, lo dejamos de lado, él va aprendiendo solito [...] (P9)*, puede perjudicar el desarrollo y la evaluación del funcionario a lo largo del año. La necesidad de mejorar el trabajo en equipo, aliada al cuidado con del otro, es identificada a veces de forma vehemente *[...] no comprendiendo a las personas como personas por falta de tiempo, [...] eso no es una prioridad porque tenemos otras demandas (P12)*, siendo un factor importante en la búsqueda del establecimiento de vínculos de confianza y calidad.

Debido a la dinámica del CQ, muchas veces el enfermero no consigue participar en el cuidado directo en el inicio del procedimiento, *[...] estar junto en la hora de la intubación del paciente, del posicionamiento, entonces cuando tú entras en la sala y ya comenzó la cirugía (P5)*, lo que puede ser un factor de frustración, ya que ésta también fue apuntada como una de las actividades que más genera satisfacción.

### La dinámica del trabajo en el centro quirúrgico no representa desgaste físico

Los enfermeros no reconocen la exigencia de la Demanda Física como influente en la percepción de la carga de trabajo, siendo más frecuente en la atención de cirugías de gran porte y complejas, como el trasplante, *[...] yo no tengo sensación de salir cansada físicamente, a no ser cuando yo tengo que atender un trasplante y quedo dentro de la sala exclusivamente. En esa situación específica, yo salgo cansada físicamente, pero sintiéndome muy realizada [...] (P3)*.

El desgaste físico es apuntado en la condición de asumir varias salas quirúrgicas, cuando hay disminución del cuadro de personal, como manifestado *[...] está con menos enfermeros, la [...] demanda física es bien mayor también (P4)*. Otro participante afirma que, por más que el CQ represente *una área muy grande aquí, [...] no necesita estar trasladándose para otros lugares, [por tener]* una área *física limitada…. los procedimientos de enfermería [...] no son [actividades] agotadoras (P6).*

### La dualidad entre el tiempo de experiencia en el centro quirúrgico y la percepción de la carga de trabajo

La experiencia puede contribuir para una mayor percepción de la carga de trabajo por tener una mejor visión de las necesidades del sector y de la institución, mayor participación en procesos y comisiones, debido a la calificación y reconocimiento por el equipo como siendo una referencia en la resolución de determinadas situaciones, conforme expresado en los siguientes trechos: *[...] las personas que están a más tiempo acaban siendo referencia, no digo especialidades, pero para algunas funciones (P1) [...] cuanto más experiencia tenemos, vamos teniendo más responsabilidades, [...] yo percibo que no solo la edad, pero el tiempo de casa, la contribución dentro del sector (P5).*

En contrapartida, algunos profesionales con más experiencia relataron menor percepción de la carga de trabajo, ya que *con el pasar de los años, va aprendiendo, [...] no dejar eso influenciar tanto en el trabajo (P1)*, por ya haber alcanzado un *nivel de relacionamiento con el equipo quirúrgico, no digo de intimidad, pero de confianza, de credibilidad del trabajo con ellos (P3).*

La inexperiencia fue apuntada como un factor que puede contribuir para menor percepción de la carga de trabajo por la disposición física, *porque ellos tienen energía y voluntad (P11), y alta capacidad de adaptación a situaciones (P12)*, o por no conocer las rutinas, los problemas y no prever ocurrencias evitables, como un factor que puede aumentar esa percepción por *no saber lidiar con lo imprevisto o [...] no tener pericia suficiente para lidiar con las intercurrencias (P2).*

### Factores organizacionales e individuales que impactan la percepción de la carga de trabajo

El nivel de envolvimiento personal, así como el no envolvimiento de compañeros aumenta el sentimiento de sobrecarga de trabajo, *[...] porque, si es una persona que sabe ocupar su lugar, que sabe la diferencia que hace, va a envolverse en más frentes de trabajo (P3)*.

El acúmulo de actividades administrativas provoca el desvío de la función asistencial del enfermero, asumiendo demandas que podrían ser realizadas por un equipo administrativo: *[...] estamos perdiendo aquello que era lo esencial de la asistencia para hacer las otras cosas, entonces nuestra carga está aumentando en razón de eso. Los flujos, los procesos que no están bien alineados, normalmente, sobran para el enfermero. [...] el enfermero también no sabe decir no, y tú no vas a dejar al paciente esperando, va a recibir un material cuando no es para recibirlo, vas a la farmacia a buscar una cosa [...], vas atrás de una factura de una otra cirugía que nadie vio… (P5)*.

La administración del equipo y adecuación del cuadro de personal fue apuntado como un factor que contribuye para el aumento de la percepción de carga de trabajo, en uno de los hospitales, conforme a los relatos: *[...] a la falta, de los certificados de los funcionarios, del dimensionamiento, de la demanda del enfermero, de la sala de urgencia, de tener cirugías más complejas, y nosotras no tener el aumento del cuadro funcional (P7)*. Por otro lado, en otro hospital fue relatado que la completud del cuadro de los enfermeros contribuye para una menor percepción de la carga de trabajo, ya que *[...] el dimensionamiento de personal mejoró bastante (P8) [...] porque, por más que tuviese bastante cosas para hacer, hoy, tenemos más enfermeros. Cuando yo quedaba sola, pensaba que mi carga de trabajo era mucho mayor, porque tenía que tener vacaciones, la escala mensual, tenía que hacer todo [...] y hoy soy responsable por una escala, todavía no consigo acostumbrarme con eso (P10).*

La falta de reconocimiento manifestada por enfermeros cuando discurren que al tornarse apenas más un número puede contribuir para hacer invisibles los esfuerzos dedicados por ellas, ya que *[...] si tú te vuelves más un denominador común así y a veces por la alta demanda, tú no consigues a veces estar tan aparente a punto de tú tener un reconocimiento. Entonces esa falta de reconocimiento a veces genera una frustración (P12).*

La exhibición conjunta e integración de los datos fue presentada en *joint display* siguiendo la propuesta de conexión, en donde los hallazgos cualitativos buscan explicar y complementar los datos cuantitativos (Figura 3).

**Figura 3 -:** Integración de los datos del índice global de la escala NASA-TLX, tiempo utilizado en la realización de las actividades y percepción de la carga de trabajo por enfermeros del centro quirúrgico. Porto Alegre, RS, Brasil, 2023

*CUAN = Cuantitativo; ^†^cual = Cualitativo; ^‡^NASA-TLX = *National Aeronautics and Space Administration - Task Load Index*; ^§^m = Media; ^||^DE = Desviación Estándar; ^¶^h = Horas; **min = Minutos

## Discusión

La Demanda Mental fue la dimensión que obtuvo la mayor puntuación en la escala NASA-TLX, reflejando como los enfermeros perciben que esa dimensión es la más desafiadora. Las razones por las cuales los enfermeros perciben la influencia de esa dimensión, en la carga de trabajo, se vuelven más claras con los datos de las entrevistas que relatan las actividades más influentes: la planificación de las escalas de personal y de flujo de la escala quirúrgica, la administración del sector y realización de la evaluación del paciente y su documentación. Esas actividades también fueron las que exigieron la mayor parte del tiempo de esos profesionales. Esos hallazgos convergen con estudios, que apuntan que el papel administrativo ejercido por los enfermeros, en el CQ, se concentra principalmente en la organización del sector y en la dinámica de los flujos de trabajo ^(2 , 28 , 35)^.

En los CQ observados, las actividades no programadas estaban relacionadas principalmente a la reasignación de sala, debido a: atrasos y/o cancelaciones quirúrgicas; reubicación de funcionarios para adecuación de las salas; y, bloqueo para atención de urgencias y trasplantes. La imprevisibilidad y la dificultad de mantener la planificación, así como el acúmulo de múltiples y concomitantes actividades, centradas en el profesional enfermero, pueden sobrecargar a esos trabajadores ^(35 - 37)^. Un estudio realizado en unidades de internación destaca que las actividades no programadas son predictoras de la carga de trabajo mental ^(38)^, lo que converge con las percepciones de los enfermeros, reflejadas en las características dinámicas del CQ.

La prevalencia del tiempo utilizado en la evaluación del paciente y documentación (16,67%) fue inferior al resultado encontrado en un estudio norteamericano, en el cual los enfermeros utilizaron 27% del tiempo en actividades de registros de salud ^(39)^. La percepción de que esa actividad impacta en la demanda mental, converge con resultados de estudios holandeses ^(40)^ e italianos ^(41)^, en los cuales más de la mitad de los enfermeros apuntó la documentación como una de las causas de percepción de carga de trabajo elevada, lo que puede ser asociado a la presión y al aumento substancial del tiempo consumido en esta actividad ^(38 - 39)^. Para amenizar el impacto en la carga de trabajo, se apunta que el proceso de documentación debe ser realizado de forma más integrada, con equipamientos próximos a los pacientes, contribuyendo para que el registro ocurra en momento concomitante con la atención ^(39)^, lo que puede atenuar el sesgo de memoria, y consecuentemente disminuir la presión de los enfermeros sobre la asertividad de los registros.

La dimensión con menor puntuación en la escala NASA-TLX fue la Demanda Física, lo que indica que los enfermeros no reconocen la influencia de esta dimensión en la percepción de la carga de trabajo, en comparación a las otras dimensiones, sugiriendo una capacidad de adaptarse a las exigencias físicas de lo cotidiano en el CQ. Los resultados de bajo puntaje en la escala NASA-TLX y en un menor porcentaje (15,66%) del tiempo utilizado en actividades como organización del sector y posicionamiento quirúrgico, sugieren que los enfermeros se sienten adaptados a la estructura física del sector y a las tareas que exigen esfuerzo físico. Este hallazgo se contrapone a los resultados de un estudio que destaca las consecuencias de la demanda física en la salud de los enfermeros; una investigación anterior constató que 77,7% de los enfermeros de sala quirúrgica relataron disturbios musculoesqueléticos relacionados al trabajo ^(42)^. Además, en una revisión sistemática y metaanálisis, los problemas lumbares fueron los más presentes en enfermeros perioperatorios, con prevalencia de 62% en 19 estudios ^(43)^.

Analizando la Demanda Temporal en conjunto con las informaciones de las entrevistas, se puede observar que los enfermeros sienten falta de: dedicar más tiempo a la supervisión del equipo y establecimiento de vínculos con el mismo; acompañar a los técnicos nuevos de enfermería en la institución; revisar y estandarizar las actividades de asistencia en sala quirúrgica; así como, realizar de capacitaciones del equipo. Tales hallazgos refuerzan la necesidad de realizar inversiones en educación permanente, inclusive frente a la intensa rutina del sector, ya que actualizaciones y capacitaciones, del equipo de enfermería, son consideradas esenciales para la calidad de la asistencia prestada ^(1)^.

El porcentaje de tiempo utilizado (14,58%) en actividades relacionadas a la presencia en sala quirúrgica, al soporte emocional al paciente y a la atención directa, converge con la baja puntuación de la dimensión Desempeño en la escala NASA-TLX, demostrando la menor participación del enfermero en esas actividades. Los hallazgos cualitativos contribuyen para la comprensión de esos resultados cuando los enfermeros manifiestan el deseo de estar más próximos al cuidado centrado en el paciente. Pocos estudios han avanzado en la medición del tiempo dedicado a las actividades de cuidado directo al paciente, en sala quirúrgica. Un estudio anterior, desarrollado en escenario brasileño, demuestra que los enfermeros consideran importante su presencia en la sala quirúrgica, inclusive en procedimientos de menor complejidad ^(44)^.

Los participantes informaron que cada uno de ellos supervisa una media de cinco salas quirúrgicas y consideran ese número adecuado delante de las demandas de actividades. Se sabe que adecuar el equipo de enfermería, a la demanda de trabajo, es un desafío, y su importancia se relaciona con la seguridad del paciente, siendo que el número insuficiente de personal, inevitablemente, aumenta la carga de trabajo, constituyéndose en un factor que puede provocar la ocurrencia de eventos adversos e influenciar negativamente la calidad del cuidado ^(45 - 46)^. El Consejo Federal de Enfermería ^(47)^ de Brasil recomienda la deducción de horas de enfermería requeridas en CQ por volumen de procedimientos segregados en tamaños, previendo un enfermero para cada tres salas quirúrgicas (electivas); también considera la necesidad de un enfermero exclusivo en cirugías de urgencia o de acuerdo con su complejidad. Es posible inferir que la baja actuación del enfermero (profesional con mayor nivel de formación) junto al paciente, está relacionado con el número reducido de trabajadores de esta categoría en los turnos de trabajo. Sin embargo, los hallazgos de este estudio conducen a reflexionar sobre la adecuación de la carga de trabajo no apenas basada en números, pero también relacionada a las características de la institución, en lo que se refiere a los procedimientos específicos realizados (como cirugías robóticas y trasplantes), así como a la formación y experiencia de los profesionales.

Entre los factores personales, la correlación positiva entre edad y percepción de la carga de trabajo, demuestra la tendencia de aumento de la percepción de carga de trabajo a medida que aumenta la edad. Los datos cualitativos contribuyen para el esclarecimiento de esos hallazgos, en lo cuales los profesionales con más experiencia fueron destacados como referencia en el equipo, tanto en conocimiento como en *expertise* para resolución de problemas. Contraponiéndose a eso, una revisión sistemática y metaanálisis de resultados con el mismo instrumento, identificó mayor percepción de la carga de trabajo entre los enfermeros sin experiencia, indicando que eso puede estar relacionado al desconocimiento del proceso de enfermería e inseguridad para resolver situaciones adversas ^(48)^.

Los factores organizacionales pueden impactar la percepción de la carga de trabajo, a pesar del poco tiempo empleado en actividades relacionadas a la revisión de la escala quirúrgica para el día siguiente, a la comunicación del equipo anestésico y de trasplante, los cuales fueron apuntados por los participantes como actividades que alejan al enfermero de la asistencia. La identificación y establecimiento de tareas administrativas, que pueden ser delegadas bajo supervisión de enfermeros, carece de estudios, inclusive con la utilización de recursos informatizados o inteligencia artificial, como propone un protocolo de estudio alemán, con el objetivo de comprobar y evaluar la aceptación de los usuarios (enfermería, equipo de servicio y pacientes) de la implantación de un sistema robótico-digital para aliviar la sobrecarga de actividades del servicio de enfermería ^(49)^.

Frecuentemente, los enfermeros asocian las funciones administrativas con el aumento percibido de la carga de trabajo, especialmente cuando el enfoque está en la gestión de escalas quirúrgicas, en lugar del cuidado directo a los pacientes. Sin embargo, esa visión no se resume apenas a la sobrecarga mental, más bien parece revelar aspectos más profundos vinculados a la formación y a los valores de la enfermería, históricamente priorizando las actividades asistenciales en detrimento de las habilidades administrativas, de enseñanza y de investigación. La dicotomía entre el cuidado directo al paciente y la gestión de los servicios, refleja una construcción cultural que valoriza más las prácticas asistenciales en detrimento de posiciones de liderazgo ejercidas por enfermeros. Esta condición se revela preocupante a medida en que puede incurrir en el desarrollo de la competencia gerencial del enfermero a medida que la gestión de los servicios puede ser considerada como dispensable. Este escenario puede provocar consecuencias graves para la profesión, considerando que la propia OMS ^(50)^ destaca el impacto positivo del liderazgo de enfermeros gestores en los servicios, pero también señala que esta presencia todavía es en número restricto, dificultando la realización de políticas de salud en escala global.

Se considera como limitación de este estudio el número de profesionales e instituciones participantes, lo que puede restringir la generalización de los resultados. Las características específicas de los participantes y la variedad de prácticas y abordajes pueden no reflejar la totalidad de la población en cuestión. De esa forma, se sugiere ampliar la diversidad y representatividad de la muestra en estudios futuros, buscando fortalecer la robustez y aplicabilidad de las conclusiones obtenidas.

Los resultados de este estudio representan nuevos *insights* de como las actividades realizadas y el tiempo utilizado por los enfermeros pueden repercutir en la percepción de la carga de trabajo. El análisis del tiempo utilizado en diferentes actividades tiene potencial para contribuir en la racionalización del tiempo y recursos, así como en el dimensionamiento adecuado de los equipos. Una comprensión más profunda puede ofrecer auxilios en la elaboración de estrategias para optimizar la distribución de tareas, mejorar la eficiencia operacional y promover un ambiente de trabajo más equilibrado y satisfactorio para esos profesionales. El estudio puede contribuir para las discusiones de entidades de clase y sociedades de especialidad sobre las atribuciones del enfermero en el CQ y su mayor envolvimiento en el cuidado directo al paciente, lo que irá a implicar en la revisión de la composición de los equipos.

## Conclusión

Al evaluar la carga de trabajo percibida y el tiempo utilizado en las actividades realizadas por los enfermeros de CQ, se constató mayor puntuación atribuida a la dimensión Demanda Mental, con predominio de actividades relacionadas a la administración del sector y de personas, además de actividades relacionadas a la evaluación del paciente y a la documentación. A pesar de que las tareas administrativas demandan una parcela predominante del tiempo de esos profesionales, la conexión directa con el cuidado al paciente, durante los procedimientos quirúrgicos, inclusive en menor proporción, los recompensa proporcionando satisfacción en el trabajo y propósito en su actuación. Esa dualidad entre gestión eficiente y entrega de cuidados directos al paciente, destaca la complejidad e importancia del papel de los enfermeros en el CQ.
